# Supplementary material for: Role of network-mediated stochasticity in mammalian drug resistance
Source: Nat Commun. 2019 Jun 24;10:2766. doi: 10.1038/s41467-019-10330-w (PMC6591227; doi:10.1038/s41467-019-10330-w)
Supplement: Supplementary file 1 — Supplementary Information [file 41467_2019_10330_MOESM1_ESM.pdf]

Supplementary Information:

Role of Network-Mediated Stochasticity in Mammalian Drug Resistance

Kevin S. Farquhar[1,2], Daniel A. Charlebois[1*], Mariola Szenk[1,3], Joseph Cohen[3], Dmitry Nevozhay[4,5], and Gábor Balázsi[1,2,3,5]

[1]The Louis and Beatrice Laufer Center for Physical and Quantitative Biology, Stony Brook University, Stony Brook, NY, 11794, USA

[2]Genetics and Epigenetics Graduate Program, The University of Texas MD Anderson Cancer Center, UT Health Graduate School of Biomedical Sciences, Houston, TX, 77030, USA

[3]Department of Biomedical Engineering, Stony Brook University, Stony Brook, NY, 11794, USA

[4]School of Biomedicine, Far Eastern Federal University, 8 Sukhanova Street, Vladivostok, 690950, Russia

[5]Department of Systems Biology, The University of Texas MD Anderson Cancer Center, Houston, TX, 77030, USA

*Current address: Department of Physics, University of Alberta, 4-181 CCIS, Edmonton, AB, Canada.

SUPPLEMENTARY METHODS

Linearity assessment with L1-norm and curve fitting.

To assess the degree and range of linearity, we calculated the L1-norm metric[1,2] for the mean expression levels from the mNF-PuroR and mNF-GFP dose-responses. The metric can be thought of as the distance between a mean expression dose-response and an ideal linear function, with 0 representing perfect linearity and 0.5 being the least linear[1,2]. First, the doses and means (i.e., coordinates) were rescaled so that the first point in the new coordinate system was (0,0) and the last dose and mean was (1,1). From these discrete values, we finely interpolated the means between doses with the *interp1.m* MATLAB function. Then, the area between the ideal linear function and the rescaled interpolated dose-response was calculated with the *trapz.m* MATLAB function. This integrated area represents the L1-norm metric up to each dose. We included 4 or more doses in each L1-norm metric calculation. The plots in **Supplementary Figure 5a,c** display L1-norms that are generally minimal over low to intermediate Doxycycline concentrations. The L1-norm abruptly increases once doses induce saturating means. To calculate the coefficients of determination or R-squared values of linearity for mean expression dose-responses, the mean expression data was also fit to a linear function.

Estimating switching rates after flow-sorting of mNF and mPF high- and low-expressing subpopulations.

We estimated the rates of gene expression fluctuations (switching rates) by flow-sorting of high-expressing and low-expressing mPF and mNF cells induced to the decoupled noise point (DNP) and then monitoring these sorted cells as they returned to their original expression distributions. We sorted the cells with the lowest and highest 15% of expression into separate, terminal wells corresponding to each subsequent time point of expression measurement. Using cells from these wells, we measured expression over time with a BD FACSCalibur Cell Analyzer flow cytometer at the Stony Brook School of Medicine Research Flow Cytometry Core facility. To threshold high- and low-expressing subpopulation fractions over time post-sorting, we split the expression values from each sorted sample by the median expression level of a corresponding unsorted control sample.

The mNF high- and low-sort fractions drifted over time to a value higher than the unsorted high fraction (0.5). Since the original distribution did not shift accordingly, the trend did not represent a biological phenomenon; it possibly arose from technical noise after fractioning very similar distributions. To detrend the mNF high- and low-sort fractional data, we individually fit the linear equation $mx + b$, with x equaling the time point (days), to three local time points in each curve with a clearly visible linear trend. The resulting time-dependent linear equation was then subtracted from each mNF high fraction value at each corresponding time point from the curve and then shifted upward by 0.5.

We fit the high subpopulation fractions of both high- and low-sorted cells over time using the following exponential curve, a general solution for two-state phenotype switching models[3,4]:

$$H(t) = A * \left(1 - e^{-(r+f)t}\right) + C, \quad (1)$$

where $H(t)$ is the high subpopulation fraction over time, r is the switching rate from low to high expression (rise rate), f is the switching rate from high to low expression (fall rate), A and C are constants. Fitting low-sorted cells resulted in consistent switching rate values.

Since the individual median threshold at every time point partitions the monitored unsorted, visibly unimodal distributions into two cellular states with equal cell numbers, and cell growth rates do not differ noticeably, the calculated fits for r and f from the sorted cells reflect symmetrical cell switching rates across a consistent midway boundary. Therefore, at each time point, the rise and fall rates are equivalent:

$$r = f = \frac{r+f}{2}. \quad (2)$$

The corresponding rates are listed in **Supplementary Table 1** while plots of high fractions over time are displayed in **Supplementary Figure 7.**

Copy number analysis with quantitative PCR.

We measured the qPCR reactions with the Applied Biosystems™ QuantStudio™ 3 Real-Time PCR system (ThermoFisher). We amplified the genomic DNA templates and used PowerUp™ SYBR® Green Master Mix (ThermoFisher, A25742) to obtain emitted fluorescence upon binding to double-stranded DNA. The standard reaction condition for the master mix was set on the qPCR instrument. Each reaction contained a total volume of 10 µL with 500 nM primers. Reactions were prepared as a serial dilution of each genomic DNA sample in triplicate over 5-fold changes in concentration. *PuroR* and *Vinculin* (*Vcl*) were targeted as the unknown target and known copy number reference standard, respectively. CHO cells contain two copies of *Vinculin*[5]. For each genomic DNA sample, we used the $2^{-\Delta Ct}$ relative quantification method, where $\Delta Ct = Ct_{\_target} - Ct_{\_reference}$. To assess the upper bounds of error, we chose to analyze the serial dilution concentration with the largest set of deviation between Ct values. Results indicating a single copy of each circuit per cell should overlap 0.5 in terms of relative copy number of *PuroR* over *Vcl*, with two copies overlapping 1, three overlapping 1.5, etc. Since the box plot comparison intervals (95%) for each circuit (bounded by red notch markers) overlap 0.5 but not 1 or higher, we reject the hypothesis that there is more than one circuit at the 0.05 significance level. The qPCR primers are listed in **Supplementary Table 5**.

Image segmentation and assessment of cell survival under drug treatment.

We used the motorized Nikon Ti-E microscope at a magnfication of 10x to capture phase contrast and fluorescent images every 24 hours after initial Puromycin treatment. Each individual sample consists of 2x3 image fields tiled together with 15% overlap but without active stitching (NIS Elements AR). To determine the number of cells in each field, distinct nuclear stained cells and green fluorescent cells were counted with a bright spot detection algorithm provided by the Nikon Elements AR software. A typical diameter between 9 to 12 µm and an average contrast of 22.7 was applied in each run of the algorithm. The resulting binary objects were merged with an OR gate and then the objects were morphologically separated with a structual element repeated throughout the image. Each object was then counted per image to construct the cell growth curves.

Gene expression noise and fitness model.

The phenomenological model presented in this section facilitates a conceptual understanding of the relationship between gene expression noise and fitness. Importantly, it permits investigating how the steepness of the fitness function affects the relative advantage and disadvantage of gene expression noise in different levels of stress.

We modeled PuroR protein concentration in mNF and mPF cell populations using the equation:

$$x_{\mathrm{NF,PF}} = \mu_{\mathrm{NF,PF}} + \sigma_{\mathrm{NF,PF}}\xi, \tag{3}$$

where $\mu_{NF,PF}$ and $\sigma_{NF,PF}$ are the mean (set to unity) and standard deviation of PuroR, respectively, and ξ is the normal distribution. The growth rate (fitness) of each cell as a function of PuroR concentration is modeled using a sigmoidal function:

$$g_{\mathrm{NF,PF}} = \frac{2}{1+\exp\left(-Hx_{\mathrm{NF,PF}}\right)} - 1. \tag{4}$$

The size of mNF and mPF cell populations is described as an exponential growth process:

$$N_{\mathrm{NF,PF}}(t) = N_0 \exp\left(g_{\mathrm{NF,PF}}t\right). \tag{5}$$

We investigated the effect of the steepness of the fitness function H and the noise ratio σ_{PF}/σ_{NF} on the advantage of mPF cells versus mNF cells under stress. Note that $\mu_{PF} = \mu_{NF}$ and $\sigma_{PF} \geq \sigma_{NF}$. The model predicts that the high-noise mPF cells have an advantage in high stress for all fitness functions, while low-noise mNF cells are at an advantage only for steep fitness functions in low stress and are at a disadvantage otherwise (**Supplementary Figure 1**). This result generalizes previous work that

established that low and high gene expression noise is advantageous in low and high stress conditions, respectively[6,7].

Detailed description of plasmid construction.

The oligonucleotides used in the construction of the plasmids can be found in **Supplementary Table 3**. The first intermediate plasmid pDN-D2irTNG5kwh was created by cutting pcDNA5/FRT and pDN-D2irTNG4kwh[1] with the SpeI-HF and SphI-HF restriction enzymes. The 4000 bp fragment from the first (Flp-In expression backbone) and 3400 bp fragment from the second (*hTetR*::NLS::*EGFP* construct with enhancements) plasmids were ligated together to make pDN-D2irTNG5kwh. The P2A sequence was introduced into the circuit by sequential extension PCR with primers CMV-PacI-f, SV40-AscI-BbvCI-r (flanking) and TN-P2A-r, P2A-TN-f, P2A-i2-f, P2A-i1-EGFP-f, EGFP-P2A-f (internal) and then inserting the product fragment into pDN-D2irTNG5kwh (cutting both with SpeI-HF, NotI-HF and ligating) resulting in pDN-D2irTN2AG5kwh (mNF-GFP).

Next, we built the mPF circuits. The pDN-MMa6h plasmid encoding the *rtTA* regulator was made by cutting pDN-D2irTN6kwh[1] with SpeI-HF and XhoI-HF restriction enzymes and using it as a backbone for a subsequent insertion of a PCR fragment amplified by CMV-SpeI-f and rtTA-XhoI-adv-r primers from the pTet-On Advanced plasmid (Clontech, 631069). Then, the pTRE-Tight promoter was PCR amplified from pTRE-Dual2 (Clontech, PT5038-5) with pTRE-Tight-SpeI-f and 2nd-OL-pTRE-hrtTA-r, while *rtTA* was amplified from pDN-MMa6h with 2nd-OL-pTRE-hrtTA-f and hrtTA-SalI-r, having overlap between the proximal ends. After overlap PCR extension of pTRE-*rtTA*, the intermediate plasmid pKF-P14MM5h was created by cutting pTRE::*rtTA* and the pDN-MMa6h vector with SpeI and SalI. We introduced the P2A and *EGFP* sequence with overlap PCR extension by adding P2A to pTRE-*rtTA* with pTRE-Tight-MluI-f and pTRE-rtTA-OL-2A-r and to hEGFP sequentially with 2A-OL-hEGFP-f, 2A-Bridge-OL-f and hEGFP-BamHI-r. Finally, the resulting pTRE-*rtTA*::P2A::*EGFP* construct and the pcDNA5/FRT vector was cut by MluI and BamHI, and ligated to produce the mPF-GFP circuit plasmid pKF-P14MM2AG5h.

T2A::*PuroR* was amplified from DC-RFP-SH01 (GeneCopoeia) with the T2A-f and NotI-AgeI-PuroR-r primers. To make mNF-PuroR, the PCR product was combined in overlap PCR extension with the mNF-GFP construct amplified with Intron-SbfI-f and T2A-hEGFP-OL-r. Both the product and pDN-D2irTN2AG5kwh vector were restriction digested by SbfI-HF and AgeI-HF and ligated to make the pKF-D2irTNP2AG-T2APuroR-5kwh plasmid (mNF-PuroR). The mPF-GFP construct was amplified with SacI-pCMV-f and T2A-hEGFP-OL-r, and combined with T2A::*PuroR* in overlap PCR extension. The resulting product and the pKF-P14MMP2AG5h vector were cut with SacI and NotI-HF and ligated to make the final pKF-P14MMP2AG-T2APuroR-5h plasmid (mPF-PuroR).

Density-based gating of flow cytometry data.

Flow cytometry data was exported as individual FCS files, which were then analyzed by custom MATLAB scripts. Raw forward and side scatter values were log-transformed, and then plotted as a 2-dimensional histogram. The number of bins was usually 60 unless adjusted whenever debris was unintentionally gated. The 2-dimensional histogram counts were then plotted as a contour, which further subdivided the plot with a density gate (**Supplementary Figure 36**). We chose the second from the widest contour level on average, which increased in density at the SSC-FSC coordinates harboring cellular events.

Evolutionary dynamics model.

The following pseudocode describes the stochastic population dynamics algorithm:

1. Determine D, P, and N via Equations (3) – (6) in Methods
2. **for** 1 to k realizations
3. $N'_s = N_s * \eta_1 *\text{randn}_1$; $N'_{max} = N_{max} * \eta_2 * \text{randn}_2$
4. **while** $t < t_{end}$ OR $N_{tot} < N'_{max}$
5. call ODE solver for Equation (7)

The number of cells that survive initial Puromycin treatment is given by: $N_s = P + N$. D is the number of cells that die during initial Puromycin treatment, P is the number of nongrowing persister cells, N is the number of growing nongenetically drug-resistant cells, and G is the number of growing genetically drug-resistant cells in the population. randn_1 and randn_2 are normally distributed random numbers, η_1 and η_2 the noise strength, and N_{max} the carrying capacity of the population. Noise is added to N_s and N_{max} to account for the experimental variability in the size of the initial surviving cell fraction and the size of the cell population on the day that the laboratory experiments were terminated. Cells switch between P and N phenotypes, and depending on the parameters, P cells can convert to G cells and N cells can mutate to become G cells. All parameters can be found in **Supplementary Table 2**.

Kill curves to determine optimal Puromycin dosage.

CHO Flp-in cells (parental) were passaged at 2.5 x 10^5 cells per well in a 6-well plate to establish toxicity over increasing Puromycin concentrations. Stock Puromycin solution was diluted to 10 µg/mL in media and further diluted to conduct a kill curve with Puromycin concentrations of 1, 3, 5, 7, and 10 µg/mL added a day after cell seeding. Cells were monitored every day and media was replenished every 3-4 days up to 2 weeks. We found that 10 µg/mL Puromycin was not toxic for both uninduced circuits.

Expanded details for circuit sequencing.

During the development of the humanized *TetR* (*hTetR*), an extra glycine amino acid was inserted directly after the start codon to complete a consensus Kozak sequence and thereby enhance translational efficiency[1]. Thus, the amino acid coordinates are shifted by +1 compared to the original *TetR* class B protein sequence (UniProtKB: P04483; PDB: 4AC0). The CRISP-ID algorithm inferred potential mutant alleles from mixed peaks in sequencing traces (ab1 files)[9]. Background cutoff percentages (ranging from 10-25%) were scaled to the quality of nucleotide calls. Each read was evaluated starting at 85 bp and up to 600 bp depending on the location of poor read quality. In doing so, we can distinguish genetic heterogeneity of potential subpopulations in the sequence reads from mixed peaks and minimize false positives derived from poor read quality. Primers used for sequencing are listed in **Supplementary Table 4**.

SUPPLEMENTARY FIGURES

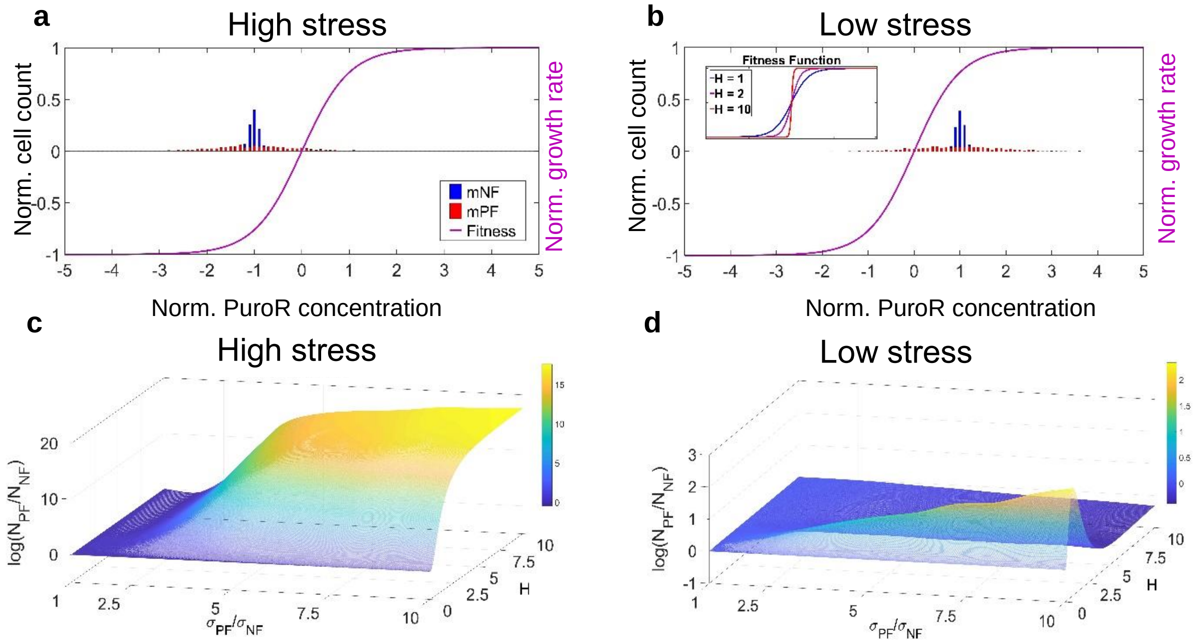

Supplementary Figure 1: The effect of fitness functions and gene expression noise on survival in a drug environment.

(a-b) Distributions and fitness function for low and high noise populations with **(a)** high or **(b)** low stress conditions. Inset shows growth rate (fitness) *g* as a function of PuroR concentration for three different values of the fitness function steepness parameter *H*. **(c-d)** Log ratio of the number of mNF (N_{PF}) to mNF (N_{NF}) cells in **(c)** high stress and **(d)** low stress conditions. Other parameters were set to: $\mu_{PF} = \mu_{NF}$ = -1 (high stress) and 1 (low stress), σ_{NF} = 0.1, σ_{PF} = 0.1 to 1, and H = 0 to 10.

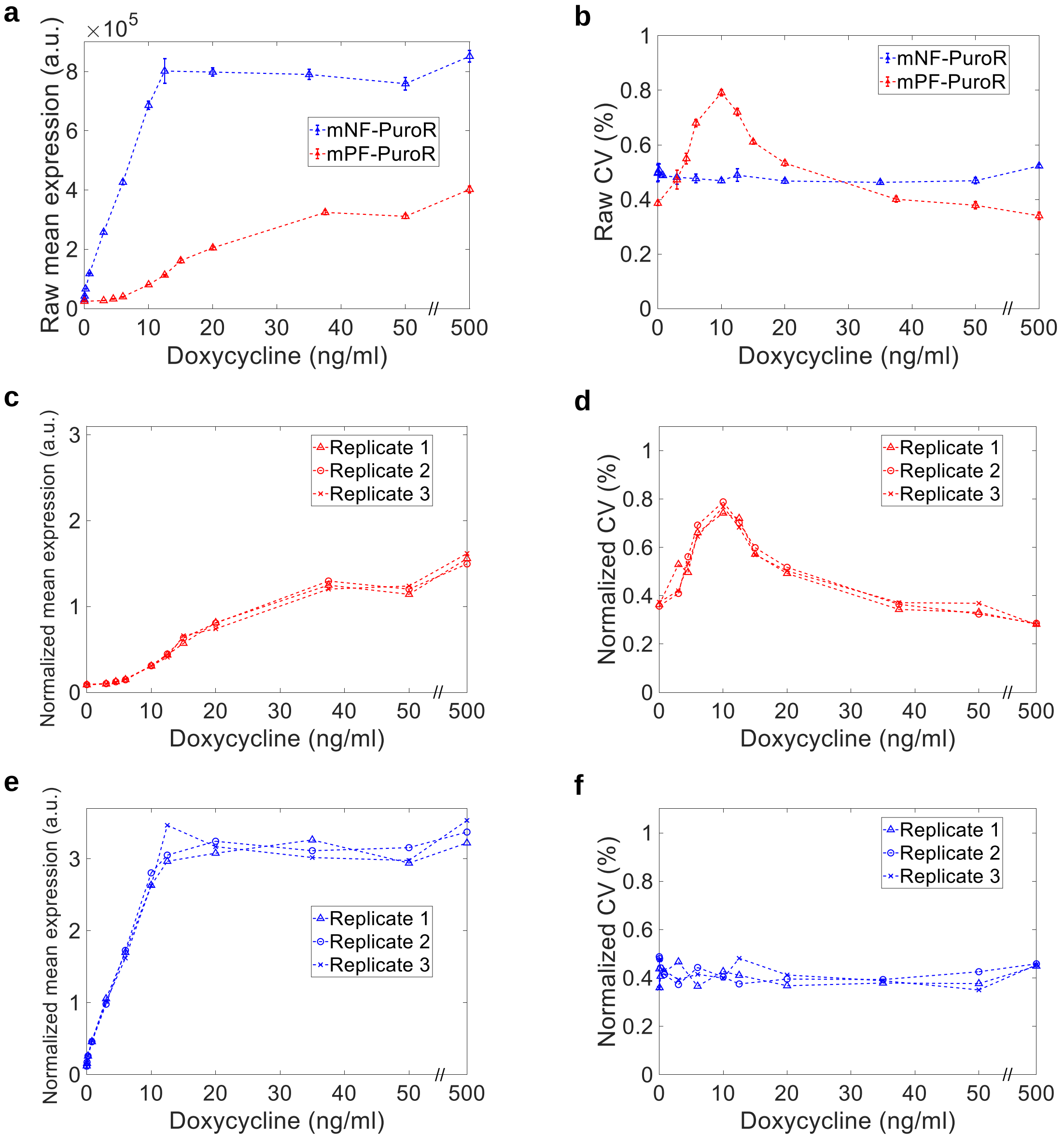

Supplementary Figure 2: Raw gene expression from the *PuroR* circuit dose-response. **(a)** Mean expression without normalization over an increasing range of Doxycycline concentrations. **(b)** Raw gene expression noise from the comprehensive *PuroR* circuit dose-response. All samples were run in triplicate. Error bars represent the standard error of the mean. **(c)** Replicate normalized mean expression for the mPF-PuroR circuit. **(d)** Replicate normalized gene expression noise for the mPF-PuroR circuit. **(e)** Replicate normalized mean expression for mNF-PuroR. **(f)** Replicate normalized gene expression noise for the mNF-PuroR circuit. Source data are provided as a Source Data file.

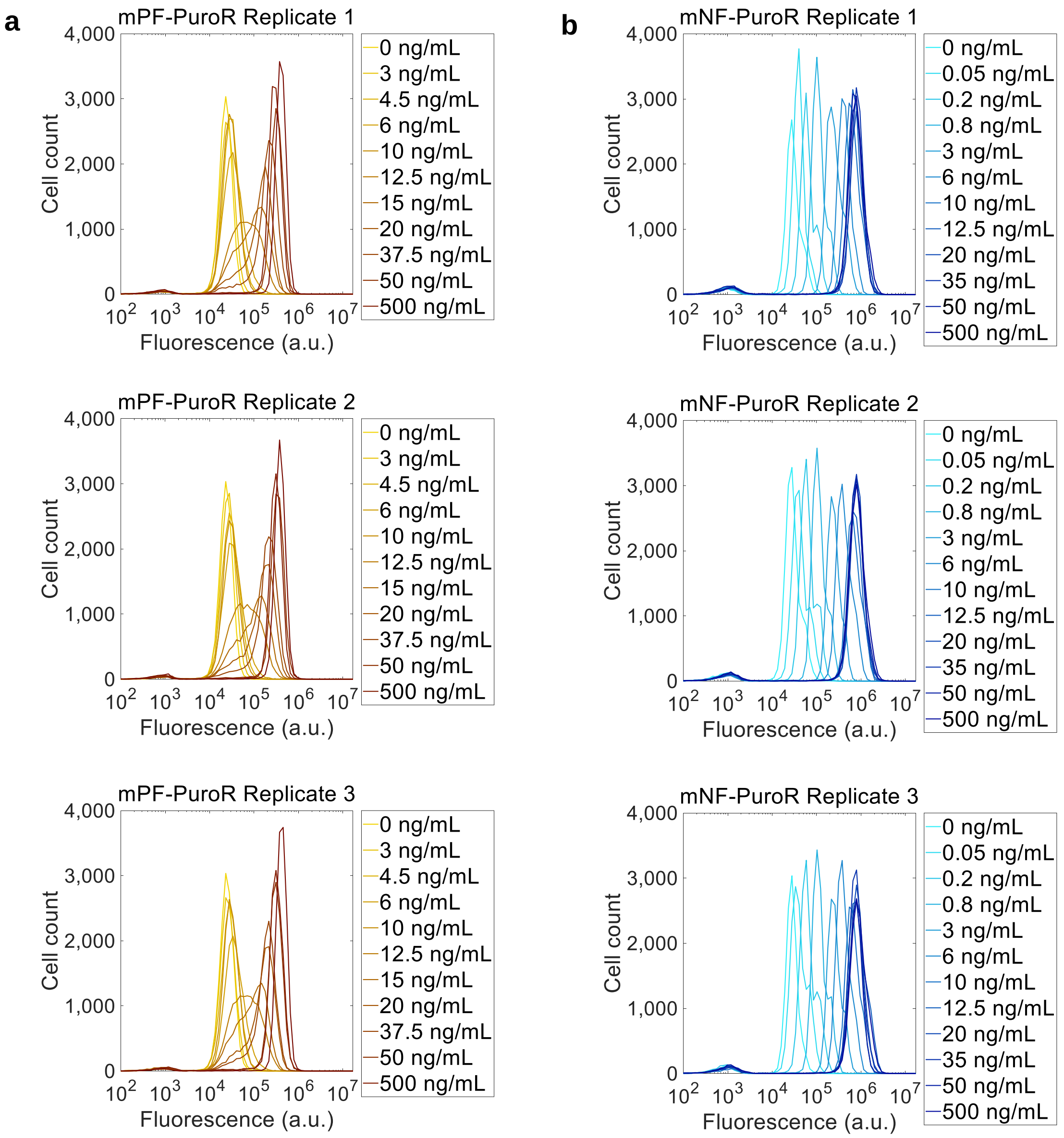

Supplementary Figure 3: Single-cell expression distributions from the *PuroR* circuit dose-response.
(a) Complete single-cell mPF-PuroR circuit expression distributions (gold to burgundy upon increasing Doxycycline concentrations). **(b)** Full mNF-PuroR single-cell expression distributions (cyan to blue upon increasing Doxycycline concentrations; see legends). A small proportion of the population from both circuits were non-expressing cells (see **Supplementary Figure 37** for sorting).

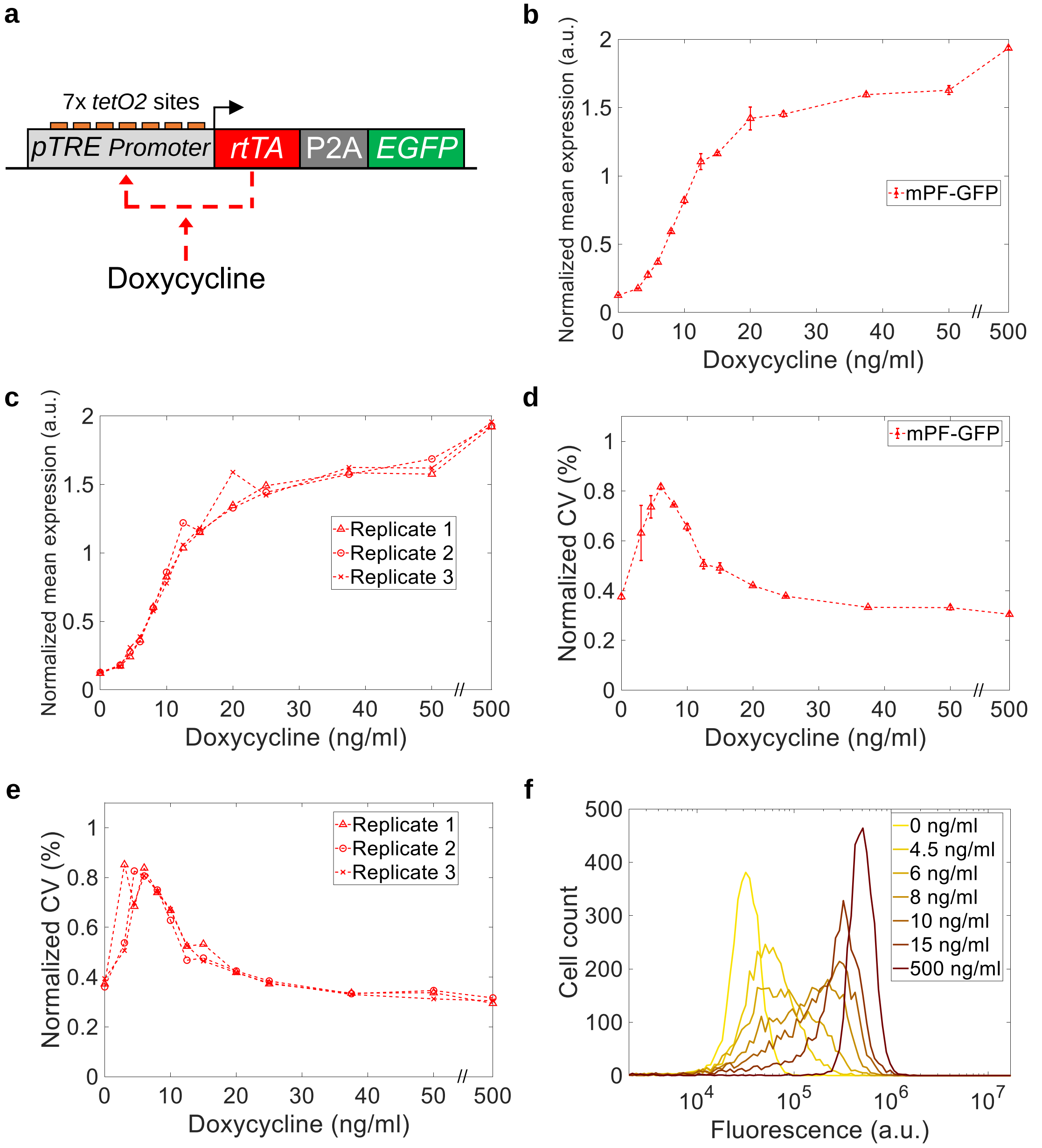

Supplementary Figure 4: The mPF-GFP gene circuit amplifies gene expression noise.
(a) The Flp-In-compatible mammalian positive feedback circuit without *PuroR* (mPF-GFP) contains the Doxycycline-inducible transcriptional regulator *rtTA* which activates its own gene expression (red arrows) by binding to *tetO2* sites in the pTRE-Tight promoter. **(b-c)** Normalized mean expression of the mPF-GFP circuit in response to increasing levels of Doxycycline in terms of **(b)** averages with error bars and **(c)** replicates. All conditions were run in triplicate. Error bars represent the standard error of the mean. **(d-e)** Gene expression noise (normalized coefficient of variation) over increasing concentrations of Doxycycline in terms of **(d)** averages with error bars and **(e)** replicates. **(f)** Single-cell expression distributions in response to increasing Doxycycline concentrations (legend, gold to burgundy). The distributions are from a representative replicate. Source data are provided as a Source Data file.

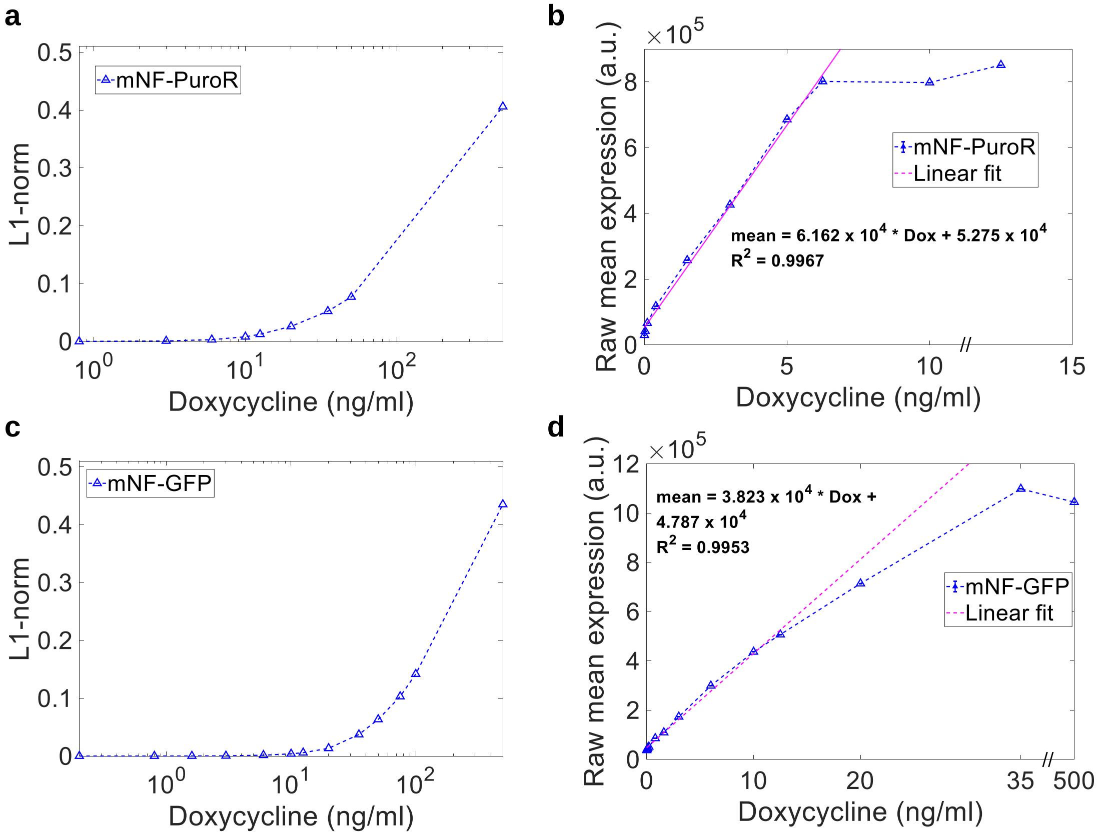

Supplementary Figure 5: Assessment of linearity for mNF-PuroR and mNF-GFP mean dose-responses.

(a) L1-norm metrics and **(b)** linear curve fits for the mNF-PuroR mean dose-response. **(c)** L1-norm and **(d)** linear curve fits for the mNF-GFP mean dose-response. The calculations incorporated average expression over replicates. Linearity is lost when expression saturates. Source data are provided as a Source Data file.

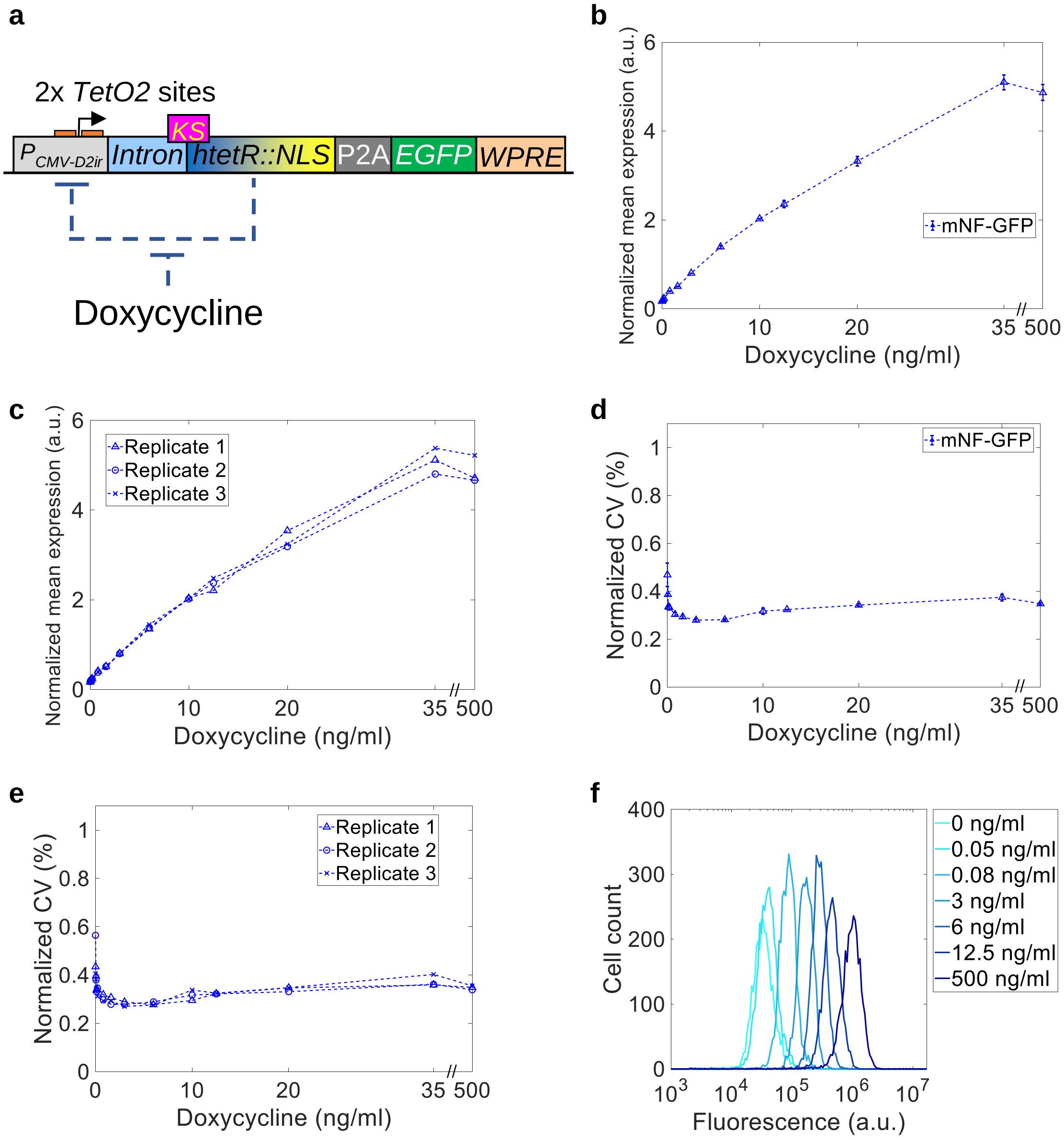

Supplementary Figure 6: The Flp-In mNF-GFP circuit uniformly expresses *EGFP*.
(a) The Flp-In-compatible mammalian NF (mNF-GFP) circuit lacking *PuroR*, engineered to linearly tune mean expression of a gene with minimal gene expression noise, contains several genetic elements intended to enhance expression, including a *beta-globin* intron, Kozak consensus sequence (KS), a nuclear localization signal (NLS) fused to *hTetR*, and a WPRE element[1]. The schematic here is more detailed than the one in the main text for mNF-PuroR, but the enhancement parts are also present in that circuit. **(b-c)** Mean expression of mNF-GFP cell populations in response to increasing levels of Doxycycline (Dox) represented as **(b)** averages with error bars and **(c)** replicates. Samples were run in triplicate. Error bars represent the standard error of the mean. **(d-e)** Gene expression noise for the mNF-GFP circuit as quantified by the coefficient of variation (CV) represented as **(d)** averages with error bars and **(e)** replicates. The data was normalized as described in the methods. **(f)** Single-cell expression distributions for the mNF-GFP circuit with increasing Dox (cyan to blue). Distributions are from a representative replicate. Source data are provided as a Source Data file.

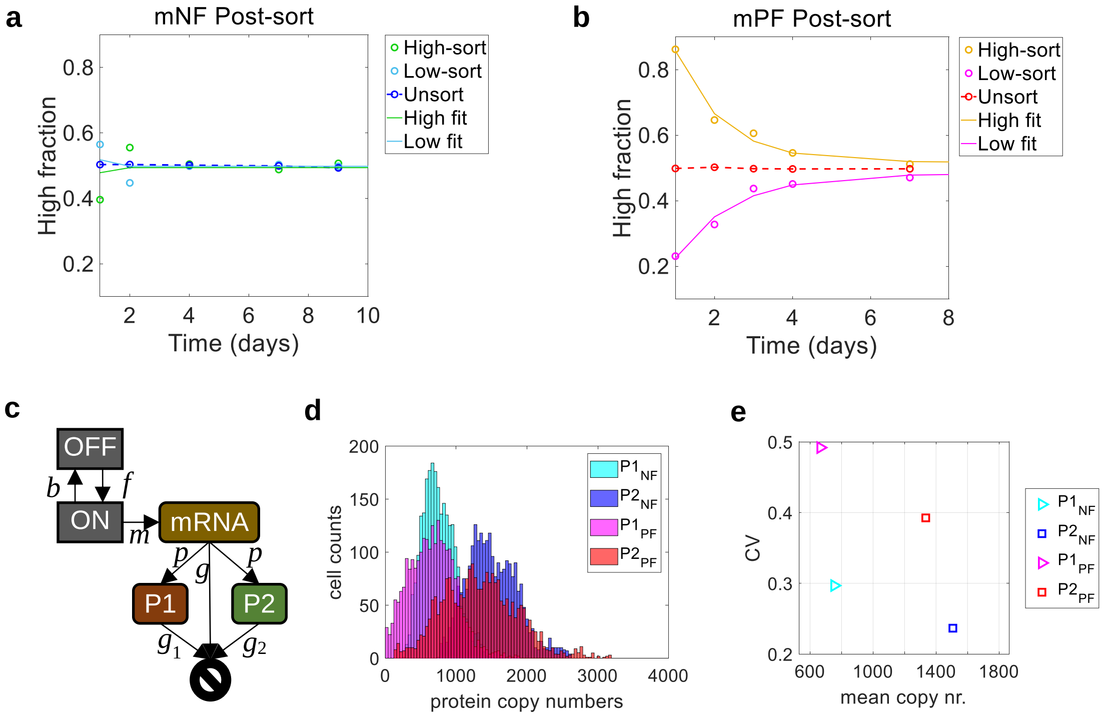

Supplementary Figure 7: Relaxation of high and low expression subpopulations after flow sorting for memory estimates and noise-mean decoupling. **(a)** The fraction of high-expressing cells for mNF-PuroR after flow sorting for high- and low-expressing subpopulations. **(b)** High fraction for mPF-PuroR after flow-sorting at the decoupled noise point. The exponential fits to the data are included in each figure. A control sample of cells was bulk-sorted without a gate as a reference for the original distribution. The mNF high- and low-sorted data was normalized as described in the Methods. Source data are provided as a Source Data file. **(c)** Simple gene expression model for testing how EGFP expression reflects PuroR expression, including noise decoupling. We set up the model in Dizzy 1.11.4 (2006), http://magnet.systemsbiology.net/software/Dizzy/ and analyzed the results in MATLAB R2018a. The parameters for NF were: $f = 2$, $b=1$, $m = 1$; $g = \log(2)/5$; $p = 10.0$; $g_1= \log(2)/10$; $g_2 = \log(2)/20$. Promoter switching rates for PF were 20 times slower; all other rates were identical. Time units are hours, concentrations are in copies/cell. **(d)** Histograms from stochastic simulations of the gene expression model. **(e)** Noise-mean decoupling for the two proteins in the gene expression model. The Matlab and Dizzy scripts can be found at https://github.com/dacharle42/MDR.

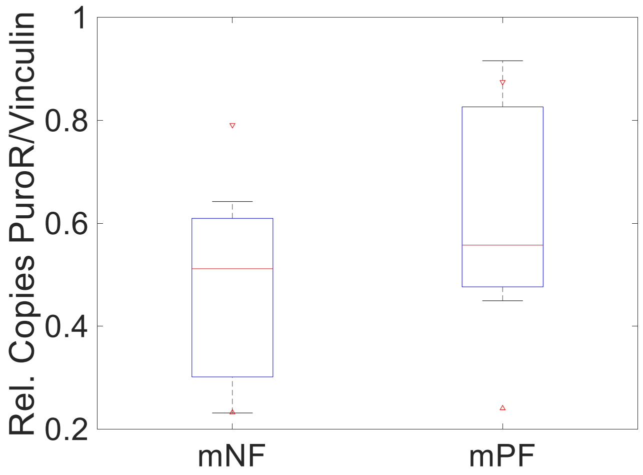

Supplementary Figure 8: Single copy integration for *PuroR* circuits verified by qPCR. Quantitative PCR results of relative copy number of the *PuroR* gene over the *Vinculin* (*Vcl*) gene (n = 3), which served as a reference for known genomic copy number (*Vcl* copies = 2). The copy number was calculated using the $2^{-\Delta Ct}$ relative quantification method. Individual reactions were conducted in triplicate (see Supplementary Methods). The median is indicated by a red horizontal line inside each box plot. The top and bottom edge of the box plots represent the 75th and 25th percentiles, respectively. The whiskers extend to the most extreme point not considered outliers. The red notch markers indicate the comparison interval end points at the 5% significance level in the case of non-overlapping intervals. Source data are provided as a Source Data file.

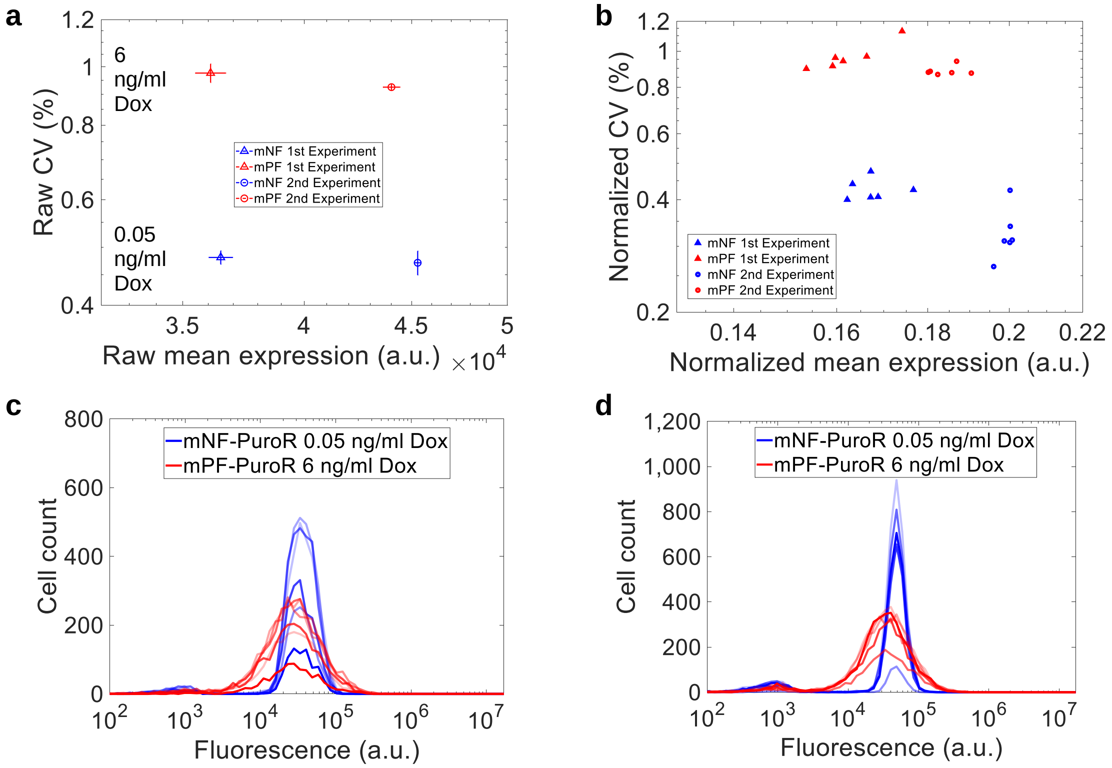

Supplementary Figure 9: Raw expression data of decoupled noise points prior to treatment.

(a) Decoupled noise points (DNPs) displaying raw mean expression and noise from the first and second experimental set. There were six replicates per sample (n = 6). The error bars represent the standard error of the mean. **(b)** Replicates at the decoupled noise points. **(c-d)** Raw expression distributions from the **(c)** first and **(d)** second experimental set (n = 6). Source data are provided as a Source Data file.

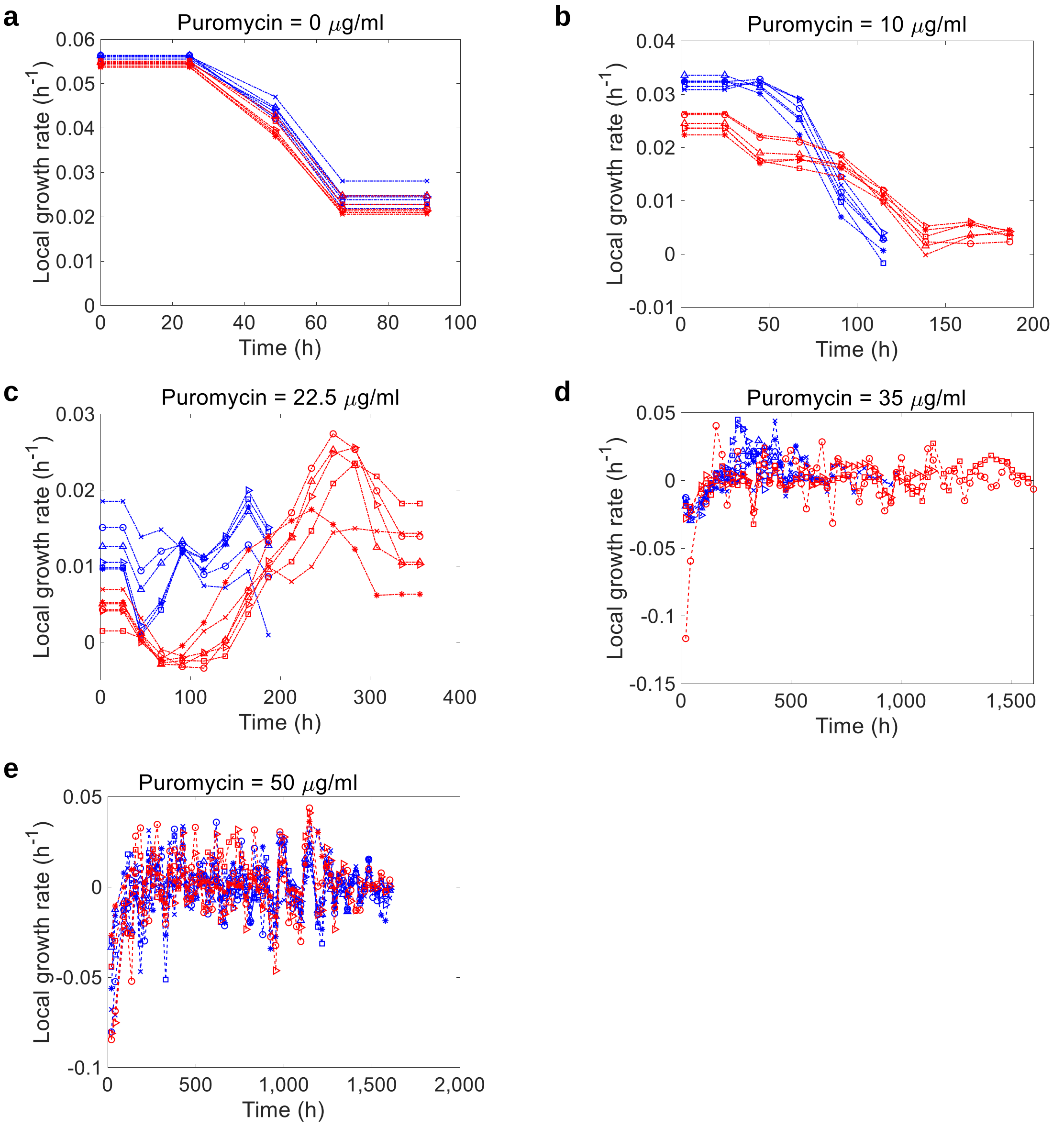

Supplementary Figure 10: Initial cell death criteria for identifying adaptation during treatment.

(a-e) Local growth rates estimated from a moving slope of 3 timepoints for 0, 10, 22.5, 35, and 50 µg/mL Puromycin. If the local growth rates dipped below 0, adaptation can occur. Each line is a replicate. Source data are provided as a Source Data file.

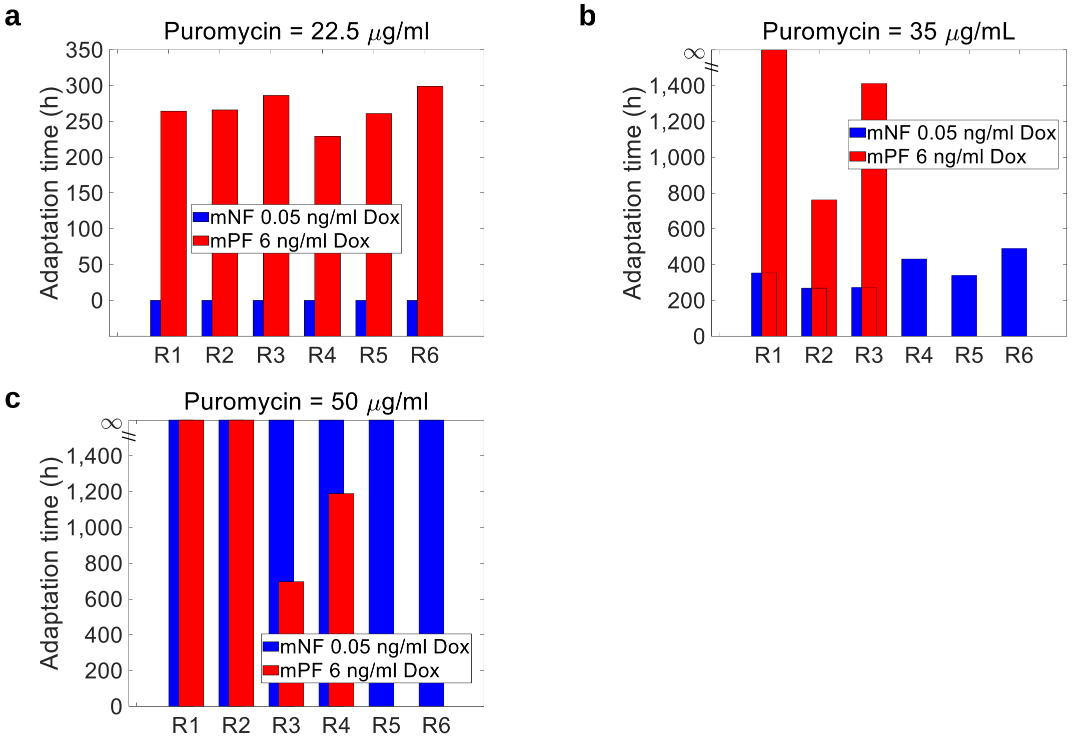

Supplementary Figure 11: Replicate adaptation times after initial treatment.
(a-c) Adaptation times for replicates with initial cell death after treatment under **(a)** 22.5, **(b)** 35, and **(c)** 50 µg/mL Puromycin. Cells quickly re-growing without initial cell death have adaptation times = 0 while treated replicates with no surviving cells have adaptation times = infinity.

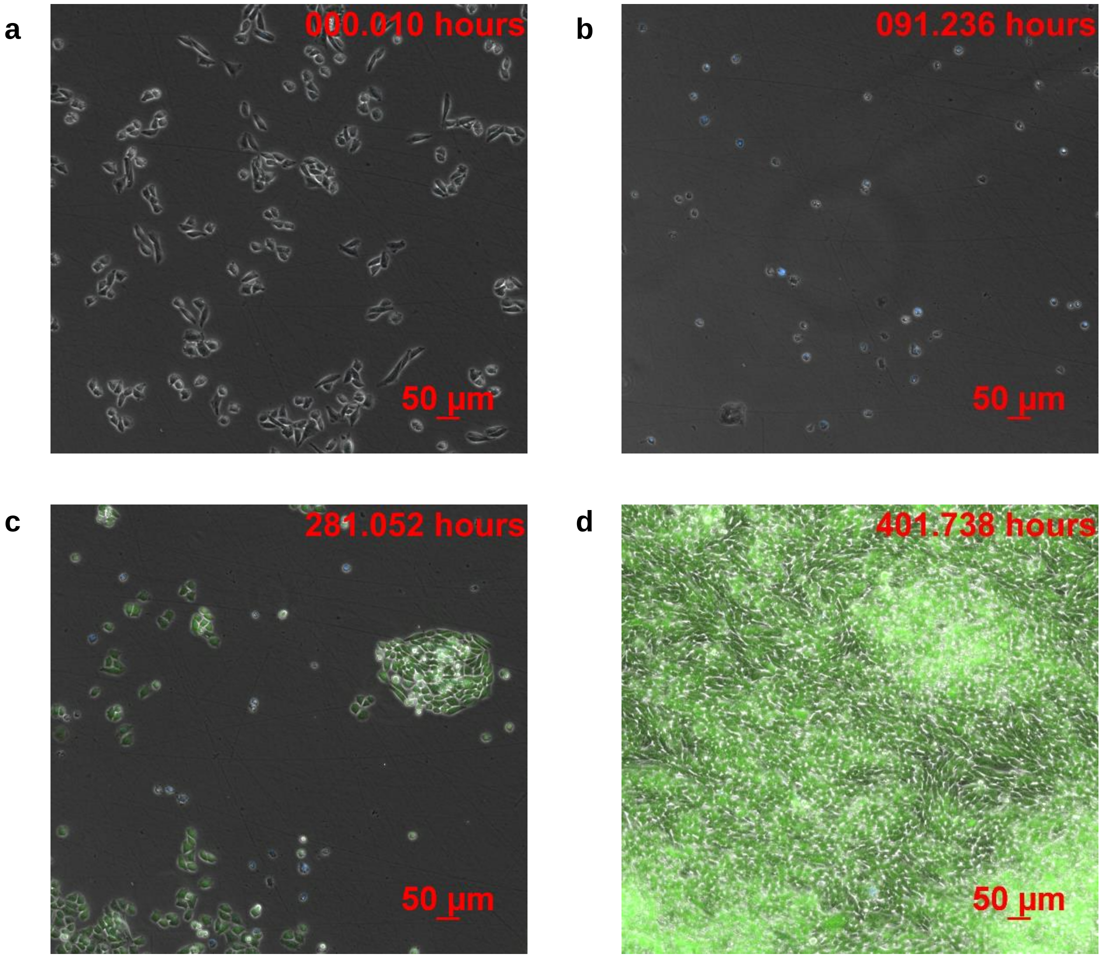

Supplementary Figure 12: Images of growth curve at various phases for mNF-PuroR replicate 2 during 35 µg/mL Puromycin treatment.
(a-d) Images at **(a)** initial treatment (0h), **(b)** growth suppression, **(c)** half-saturation, and **(d)** growth saturation phases. Time stamps are listed in the upper-right corner, which come from **Fig. 5f**. The plotted images were cropped from the raw image data with consistent look-up table thresholds for GFP and DAPI fluorescence.

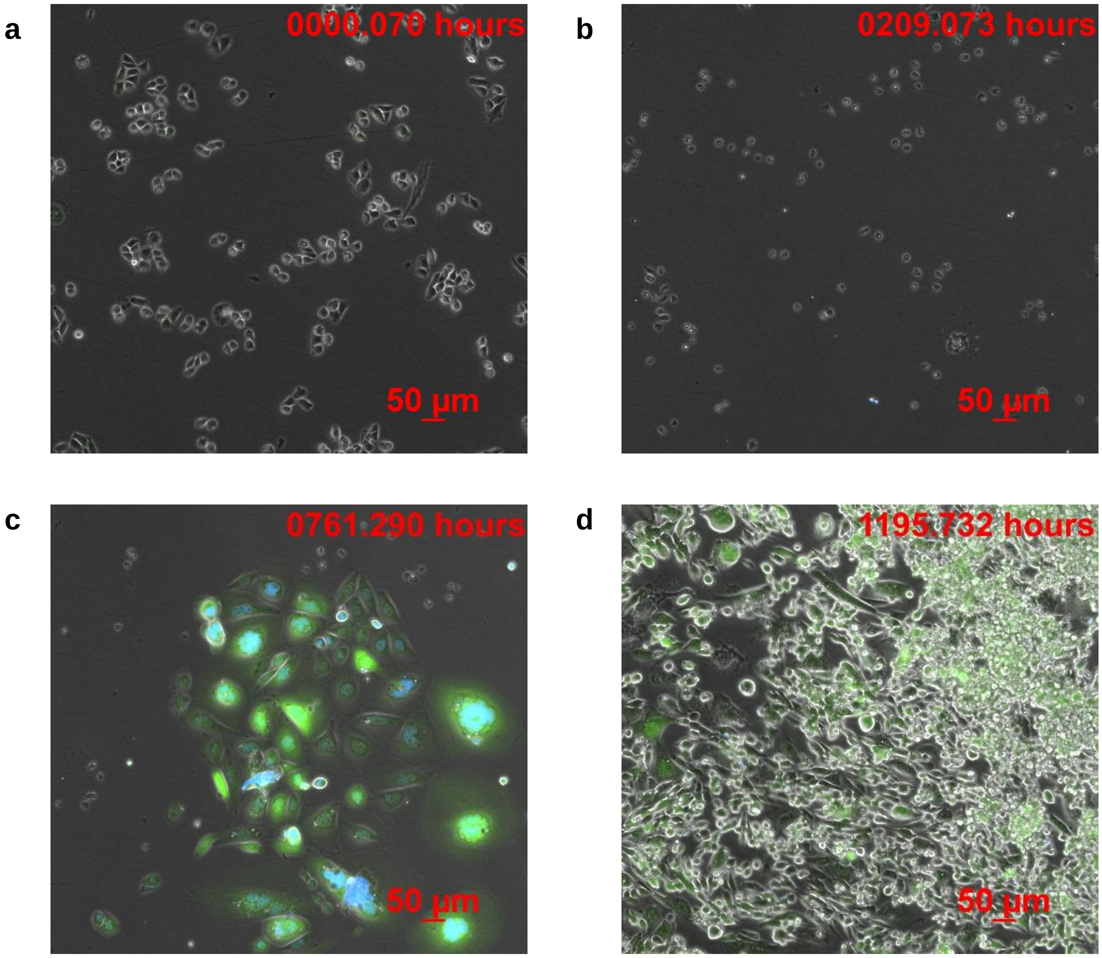

Supplementary Figure 13: Images of cells from mPF-PuroR replicate 2 during 35 µg/mL Puromycin treatment.
(a-d) Images at **(a)** initial treatment (0h), **(b)** growth suppression, **(c)** half-saturation (notice the large polynucleated cells), and **(d)** growth saturation. The times corresponding to the growth curves in **Fig. 5f** are listed in the upper-right corner. The plotted images were cropped from the raw image data with consistent look-up table thresholds.

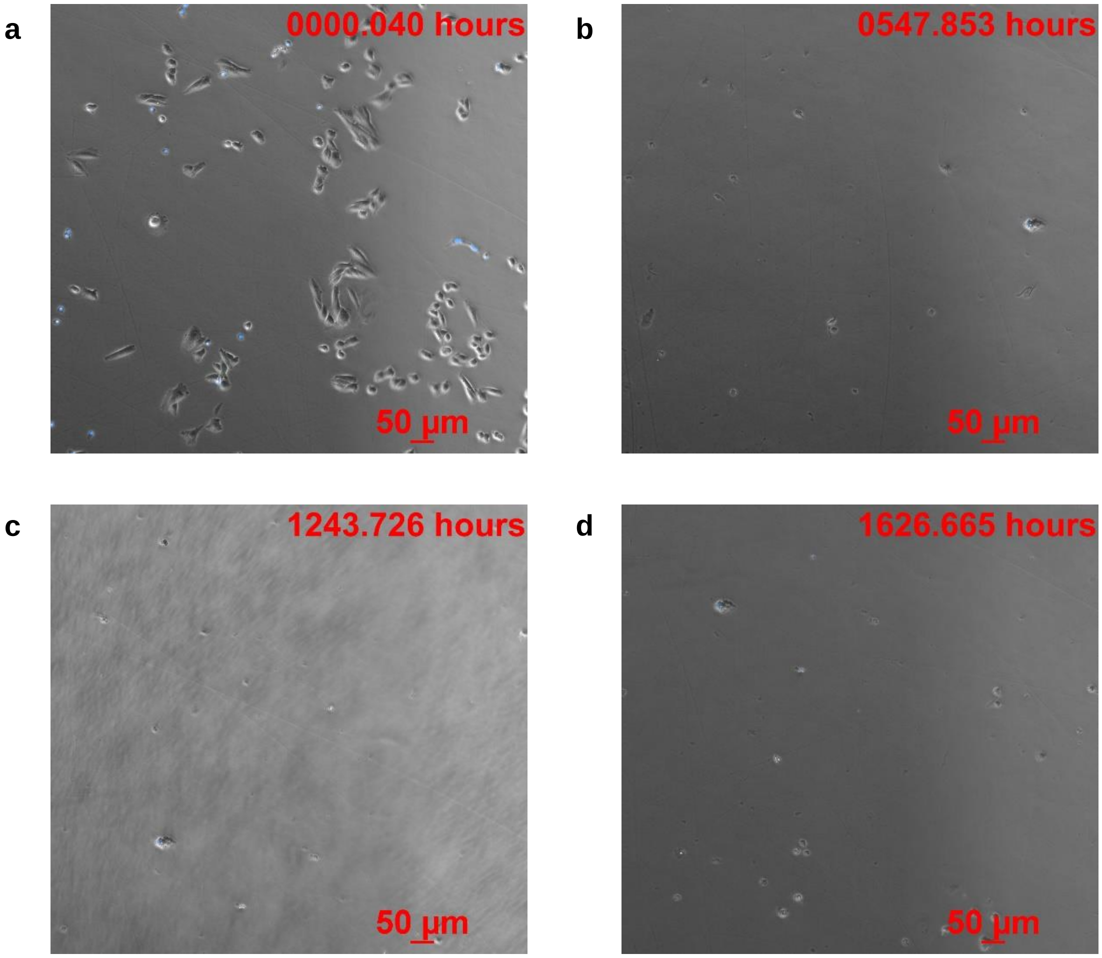

Supplementary Figure 14: Images of growth curve phases for mNF-PuroR replicate 2 during 50 µg/mL Puromycin treatment.
(a-d) Images of **(a)** initial treatment, **(b)** growth suppression, **(c)** half-saturation, and **(d)** growth saturation. The times corresponding to the lack of growth curves in **Fig. 5g** are listed in the upper-right corner. The images were cropped from the raw imaging data with consistent look-up table thresholds.

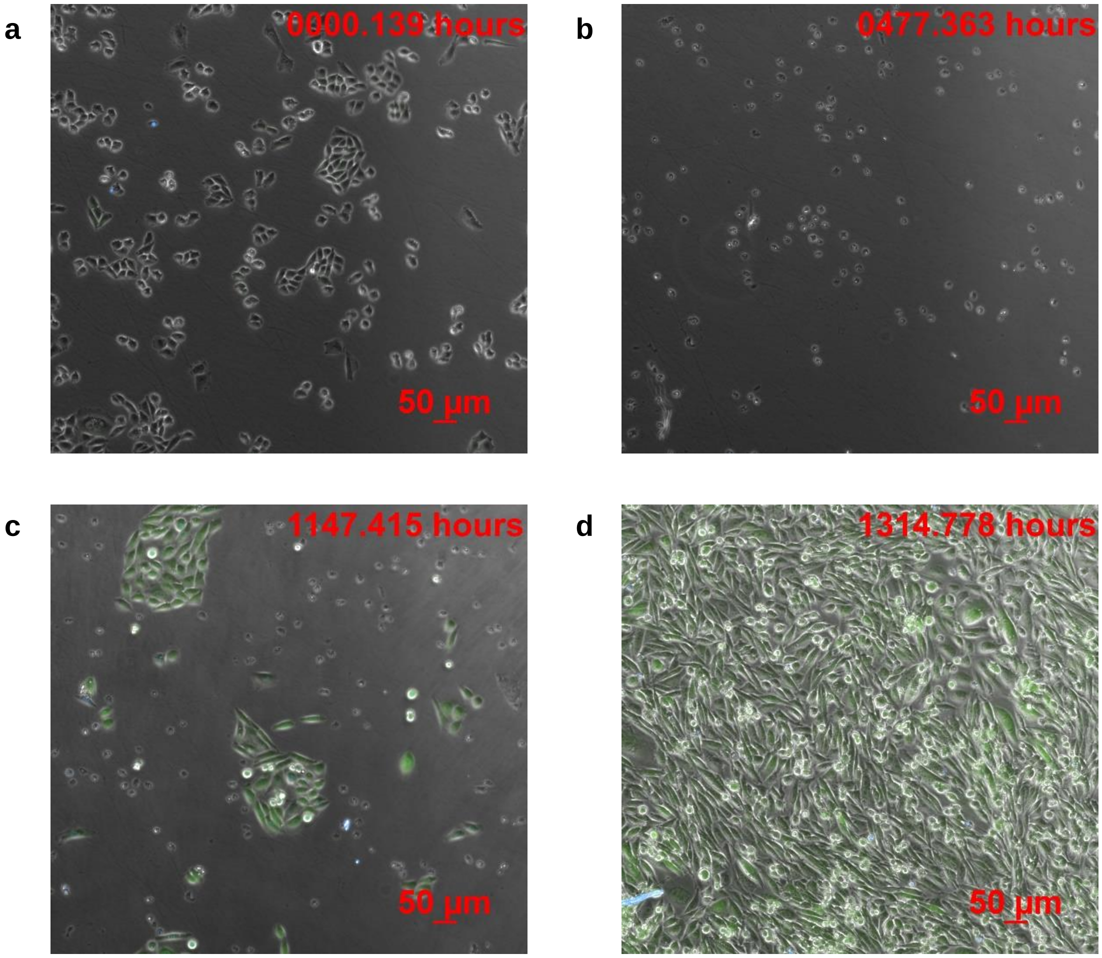

Supplementary Figure 15: Images of mPF-PuroR replicate 4 cells during 50 µg/mL Puromycin treatment.
(a-d) Images of **(a)** initial treatment (0h), **(b)** growth suppression, **(c)** half-saturation, and **(d)** growth saturation. The times corresponding to the growth curves in **Fig. 5g** are listed in the upper-right corner. The plotted images were cropped from the raw image data with consistent look-up table thresholds.

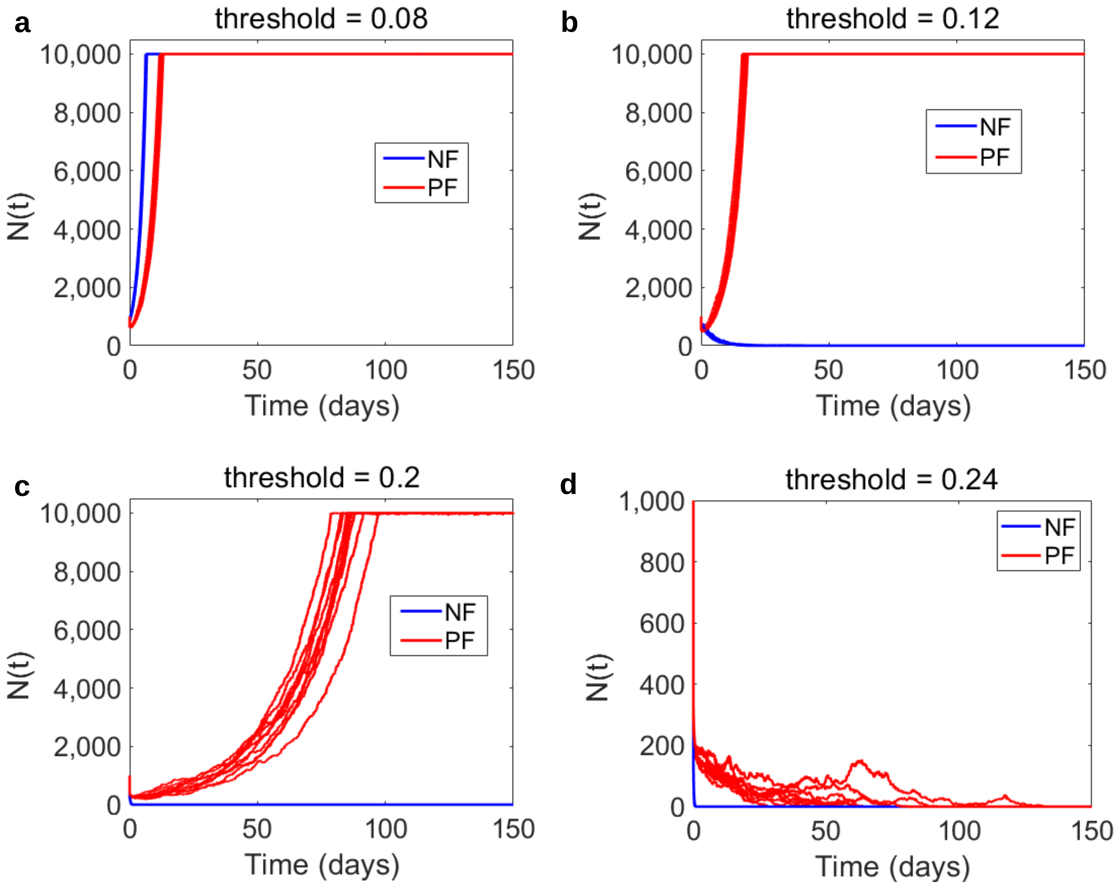

Supplementary Figure 16: Gene expression noise alone cannot explain experimental adaptation characteristics.

(a-d) Plots show cell population growth under increasing Puromycin concentrations, represented by the increasing thresholds required for survival. We set the cell growth rate (0.5 per day), gene expression fluctuation rates (2.5 per day and 0.4 per day), noise (CV = 0.5 and 1) and mean (0.16) to match the mNF and mPF experimental values. We modeled stochastic gene expression in each cell by a second-order approximation to the Ornstein-Uhlenbeck process[11], with 10,000 time steps, similar to previously used models[12,13]. All simulations results were calculated from 10 realizations of 1000 cells. The Matlab scripts *ou_second_NFPFstats.m* and *procP2A.m* can be found at https://github.com/dacharle42/MDR.

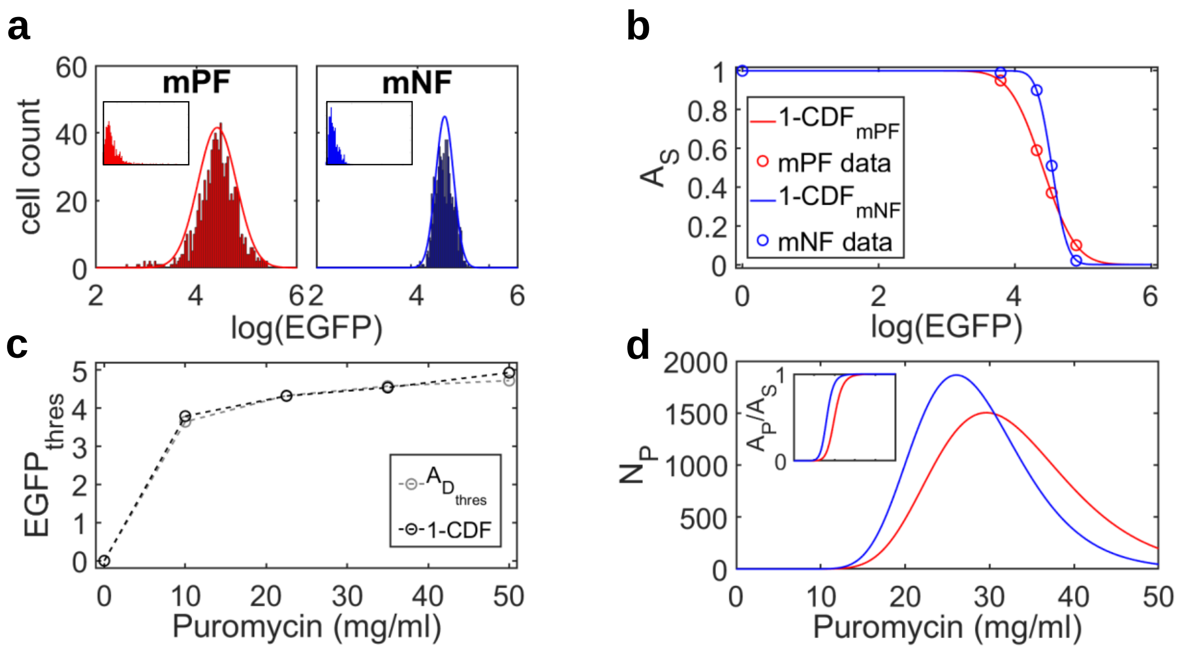

Supplementary Figure 17: Determination of initial dead, persister, and nongenetically drug resistant CHO subpopulation fractions.
(a) Fits to log-transformed CHO mPF-PuroR and mNF-PuroR gene expression data for doxycycline = 6 and 0.05 ng/ml, respectively, using Equation (3). Insets show experimental non-transformed lognormal distributions. **(b)** Fraction of cells surviving initial Puromycin treatment (A_S) calculated from 1 - Equation (4) (solid lines) along with mPF and mNF data (open circles). **(c)** *EGFP* threshold below which CHO cells are killed by Puromycin using Equation (5) (grey data points and dashed line) and the cumulative distribution function (CDF) (black data points and dashed line) from **(b)**. **(d)** The number of persister cells (N_P) as a function of Puromycin concentration. Inset shows the fraction of surviving cells that are persisters.

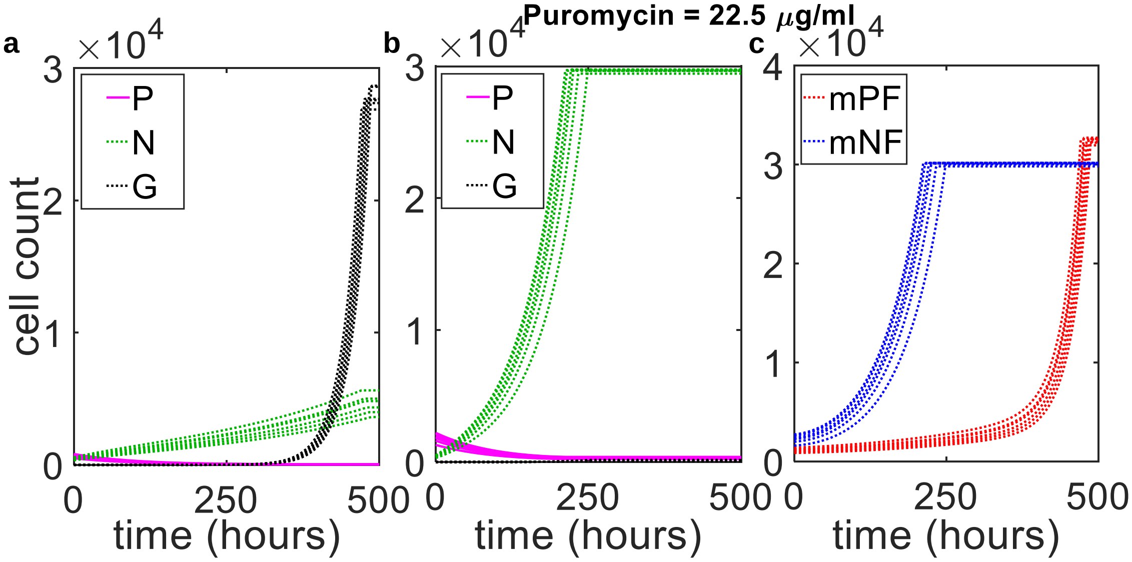

Supplementary Figure 18: Modeling the adaptation of mPF-PuroR and mNF-PuroR cells without P to G conversions in 22.5 µg/mL Puromycin.
(a) Representative growth curves for simulated mPF-PuroR persister (P, magenta lines), nongenetically Puromycin-resistant (N, green dashed line), and stably Puromycin-resistant (G, black dashed lines) CHO cell subpopulations. **(b)** Growth curves for the same simulated mNF-PuroR CHO subpopulations as in **(a)**. **(c)** Growth curves for simulated mPF-PuroR (red dashed lines) and mNF-PuroR (blue dashed lines) CHO cell populations. The model is described in the Methods, where $r_{G,P}$ = 0 for mPF-PuroR and mNF-PuroR cells and all other parameter values are as given in **Supplementary Table 2.**

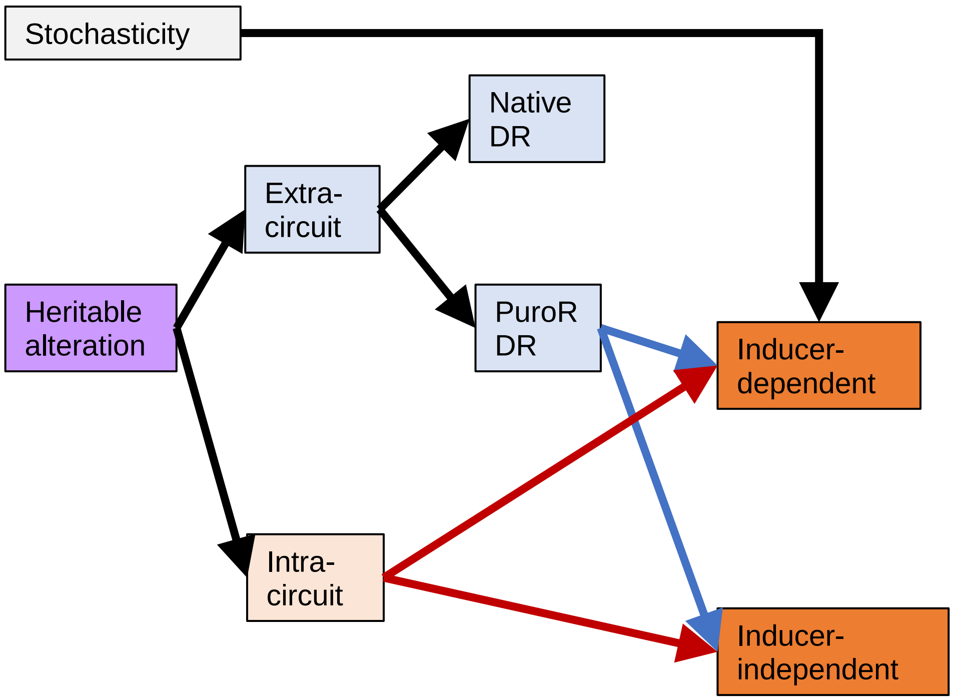

Supplementary Figure 19: Evolutionary scenarios for adaptation to Puromycin treatment. Two overarching categories of mechanism can drive drug resistance evolution in the mNF and mPF cell populations: heritable alteration (purple) and stochastic gene expression (light gray). Heritable alterations can occur either inside (tan, red arrows) or outside (light blue) the gene circuit. Extra-circuit alterations could elevate PuroR expression (i.e., be *PuroR*-dependent, blue arrows) or could elicit *PuroR*-independent, native resistance mechanisms. In these experiments, adaptation was always *PuroR*-dependent. *PuroR*-dependent adaptation could further be classified as inducer- dependent or independent (orange). Gene expression stochasticity as a resistance mechanism should be inducer-dependent, based on the noise amplitude and noise memory at the given inducer level. Inducer-dependence should be indicated by expression reversion to pre-treatment levels in uninduced populations after drug removal.

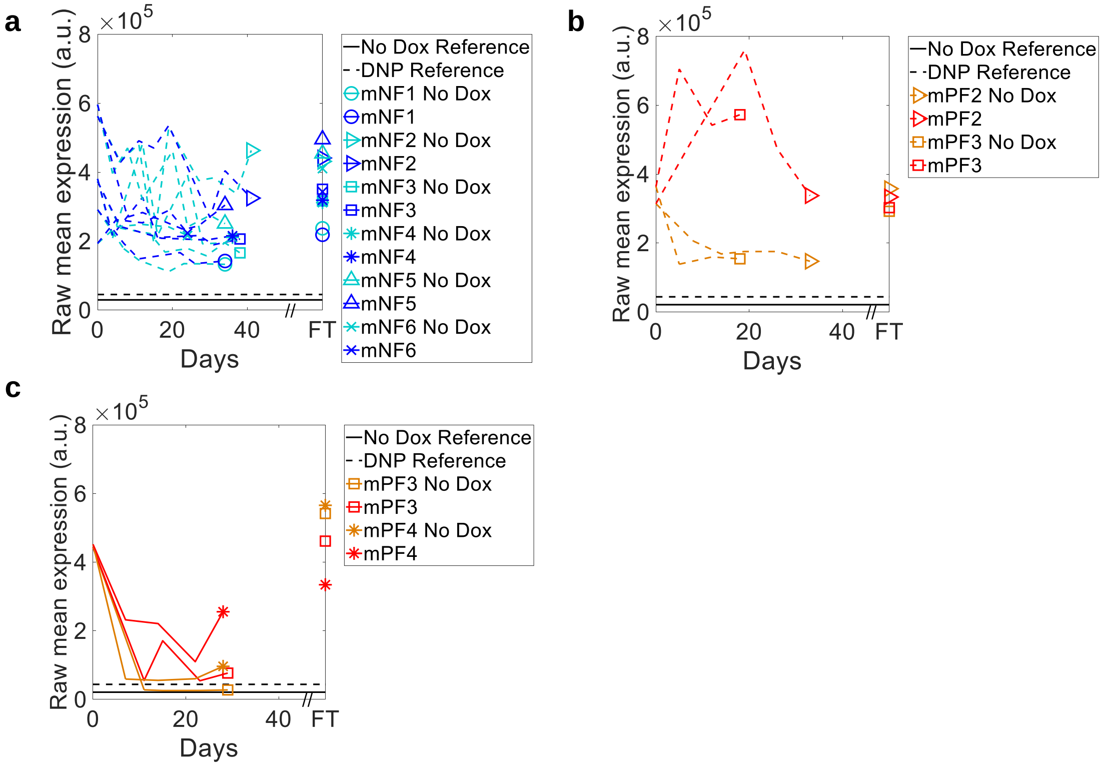

Supplementary Figure 20: Raw mean expression during the drug removal and retreatment phase for 35 and 50 µg/mL Puromycin.
(a) Raw mean expression of mNF-PuroR after temporary removal of 35 µg/mL Puromycin and retreatment. **(b-c)** Raw mean expression of mPF-PuroR after temporary removal and re-addition of **(b)** 35 and **(c)** 50 µg/mL Puromycin. Uninduced expression and expression at the decoupled noise point for each circuit served as references for successful expression resetting. Source data are provided as a Source Data file.

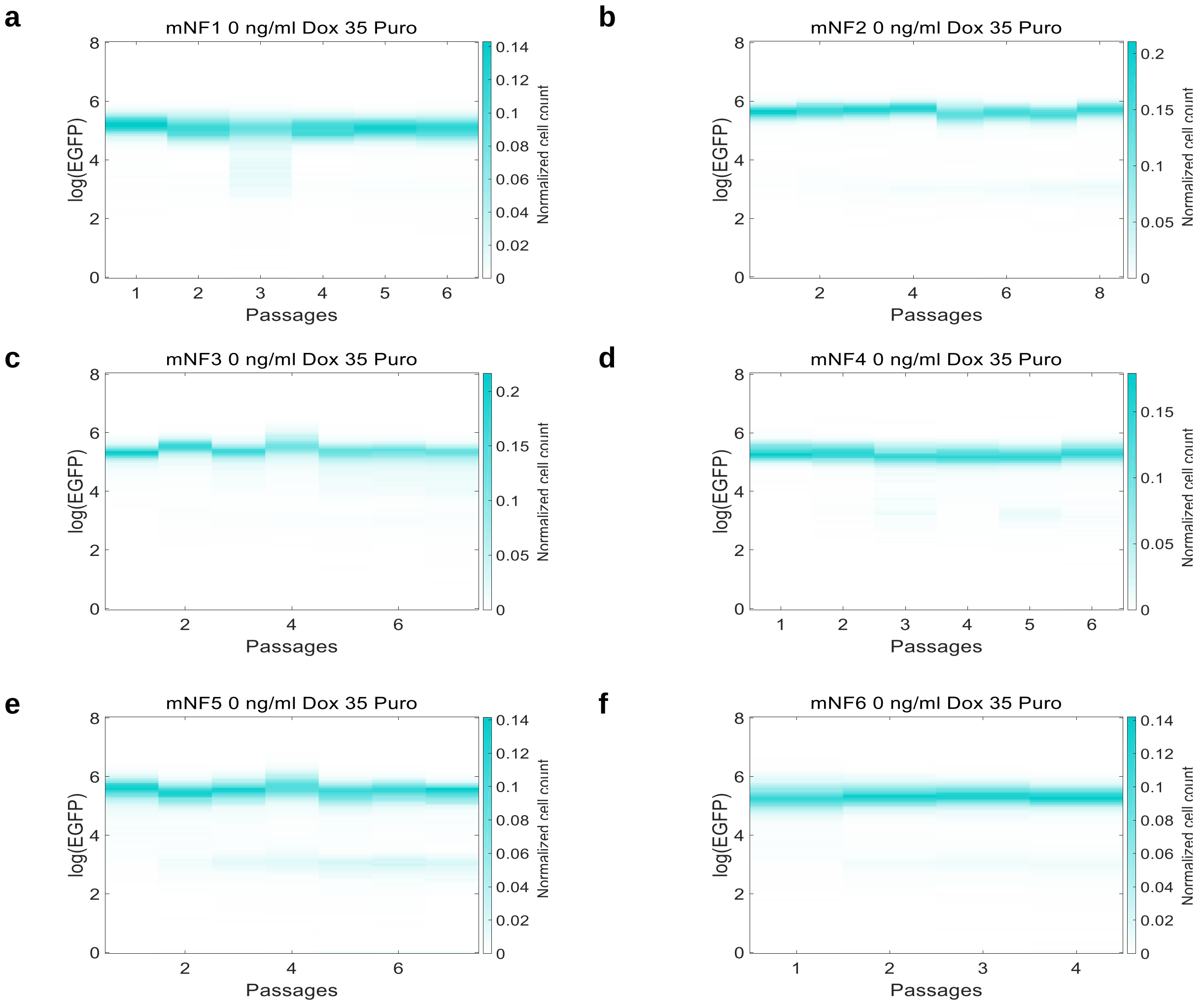

Supplementary Figure 21: Expression distributions of uninduced mNF-PuroR after removal of 35 µg/ml Puromycin.

(a-f) Full log-transformed expression distributions for uninduced mNF-PuroR replicates 1-6 after removal of 35 µg/mL Puromycin. Each color bar represents normalized cell counts gong from low (white) to high (cyan).

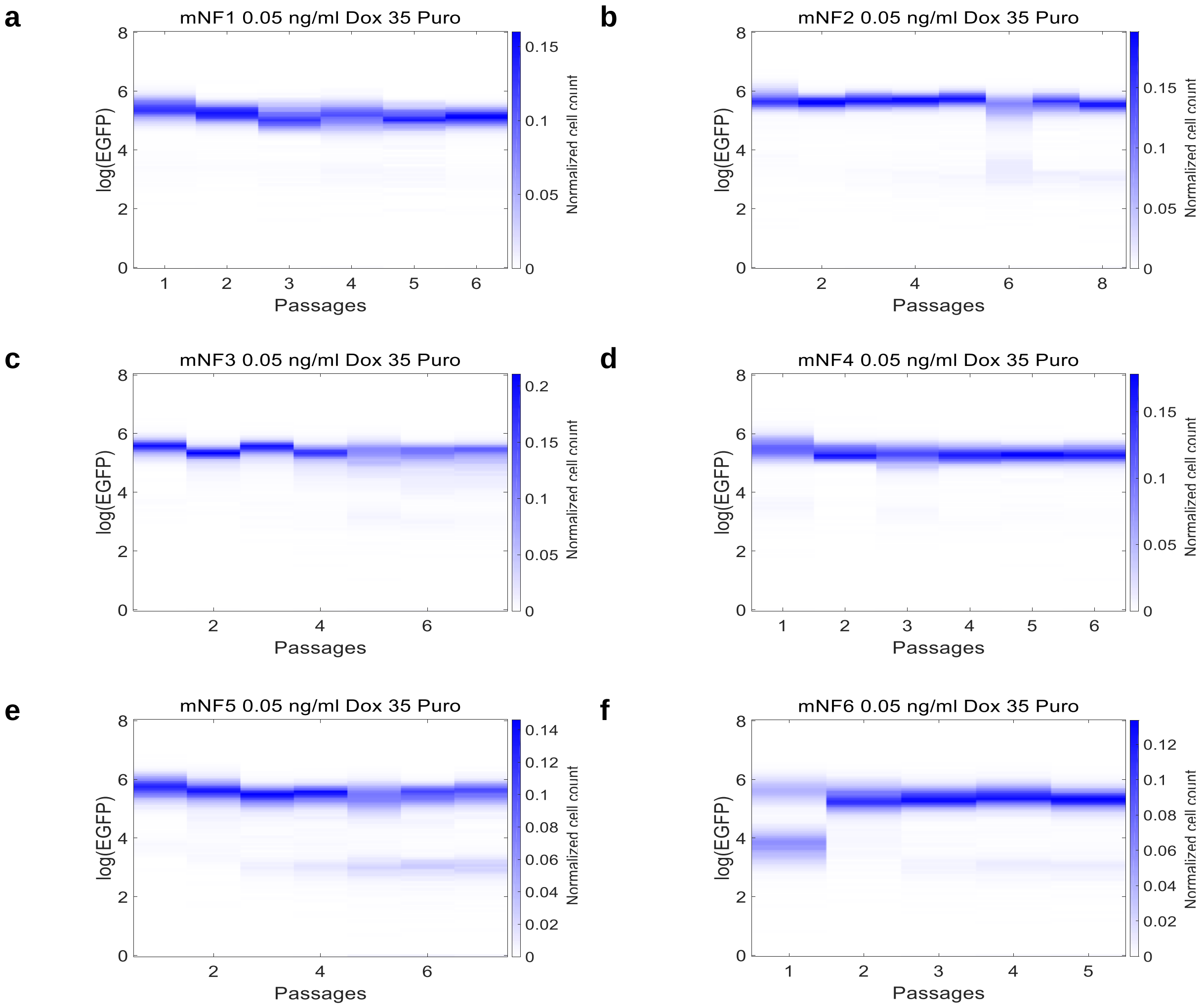

Supplementary Figure 22: Expression distributions of induced mNF-PuroR after removal of 35 µg/ml Puromycin.

(a-f) Log-transformed expression distributions for induced mNF-PuroR replicates 1-6 after removal of 35 µg/mL Puromycin. Each color bar represents normalized cell counts going from low (white) to high (blue).

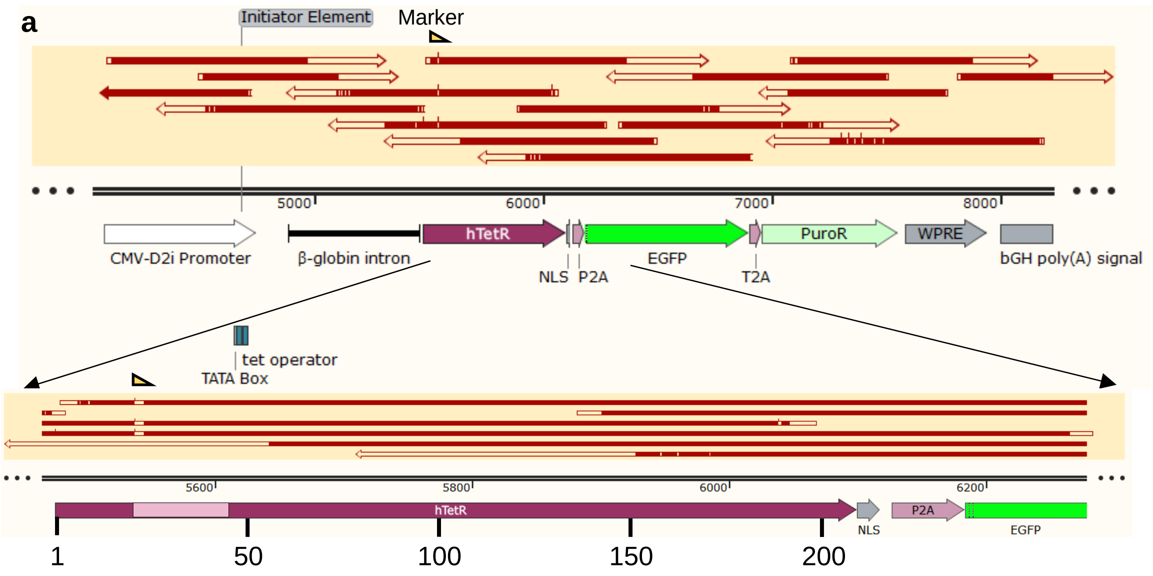

b

Replicate 2 (aa): -21–> V~~GI~~EGLTTRK 31 –> LAQKLGVEQP 41 –> TLYWHVKNKR 50 ->

α1 α2 α3

c

Reference (bp): -51-> ACTCAATGAAGT CGGTAT CGAAGGCCTGACGACAAGGAAACTCGC -96->

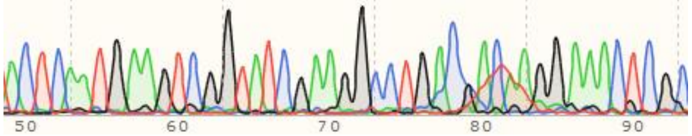

Replicate 2 (bp): -51-> ACTCAATGAAGTA~~CGGTATC~~GAAGGCCTGACGACAAGGAAACTCGC -96->

Marker

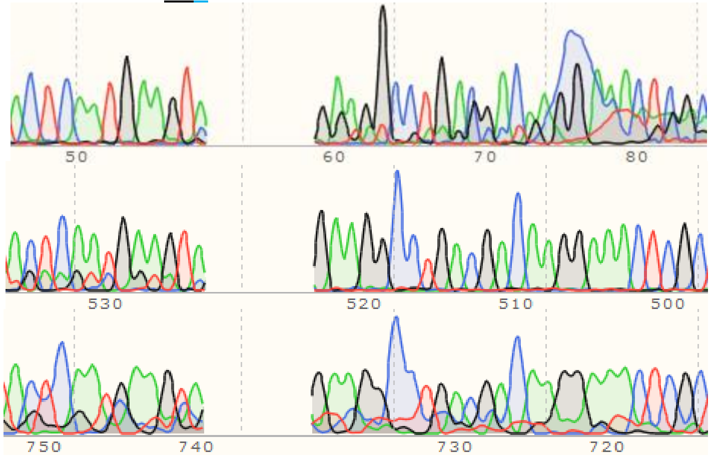

Supplementary Figure 23: Sequencing mNF-PuroR replicate 2 after 35 µg/mL Puromycin treatment revealed a DNA-binding-abolishing mutation in *hTetR*.
(a) Sequencing map (red reads) of the entire mNF-PuroR circuit from replicate 2 after 35 µg/mL Puromycin treatment. The zoom-in of humanized *TetR (hTetR)* has a lightly shaded region designating the DNA-binding domain and a marker indicating the location of a fixed mutation. **(b)** Glycine (Gly22), which is part of the first alpha helix adjacent to the hTetR DNA-binding domain (underlined), was deleted along with Ile23. Deletion of Gly22 reduces TetR protein binding affinity to *tetO2* operators by 1,000-fold[8]. Alpha helices are displayed under the corresponding amino acids. **(c)** Reference and replicate 2 nucleotide sequences corresponding to the affected amino acids (underlined). Light blue nucleotides indicate insertion events while crossed red nucleotides indicate deletions. Valine (Val21, GTC->GTA) was unaffected by the insertion. The presence of the indel is supported by three individual reads (see chromatographs).

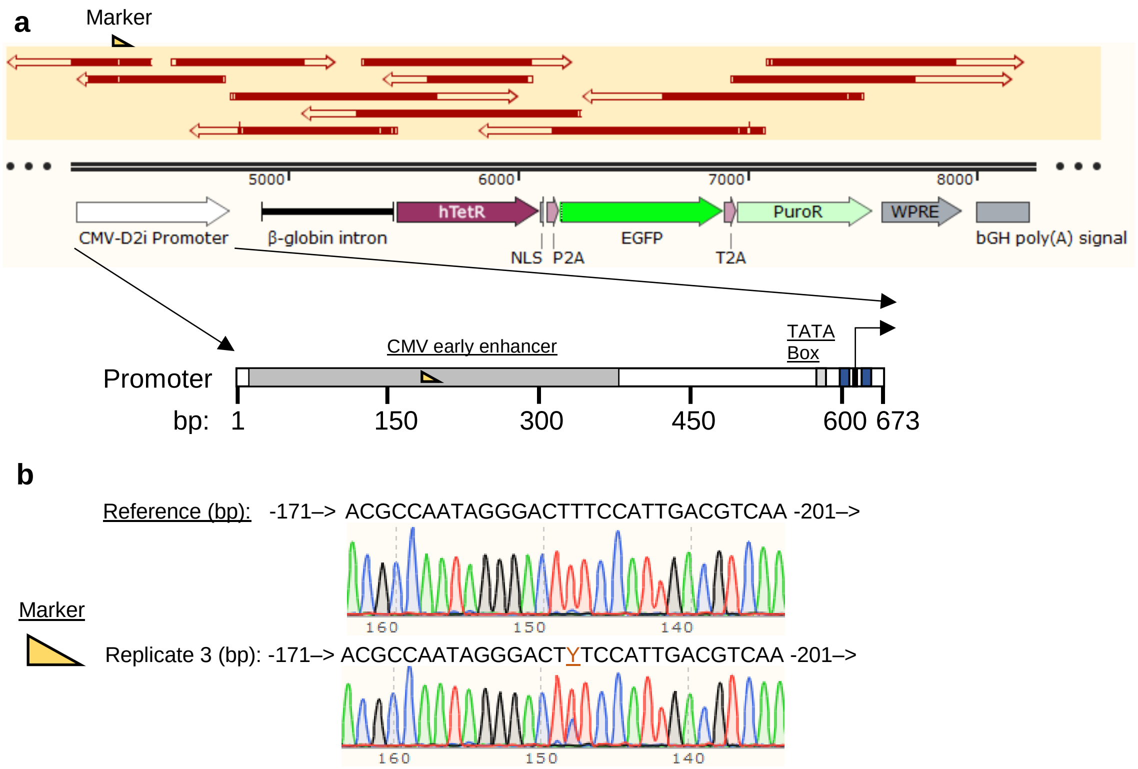

Supplementary Figure 24: Mutation in the early CMV enhancer shows evidence of genetic heterogeneity in the mNF-PuroR circuit from replicate 3 after 35 µg/mL Puromycin treatment.
(a) Sequencing map (red reads) of the entire mNF-PuroR circuit from replicate 3. The zoom-in of the promoter has multiple elements including the CMV early enhancer (dark gray region), TATA box (light gray), two *tetO2* operator sites (blue), and the Initiator (Inr) element (black). The markers indicate the location of the mutations. **(b)** Nucleotide sequence for the reference promoter and the replicate 3 promoter at the vicinity of the mutation in the CMV early enhancer at T186->Y (C/T mixture; underlined).

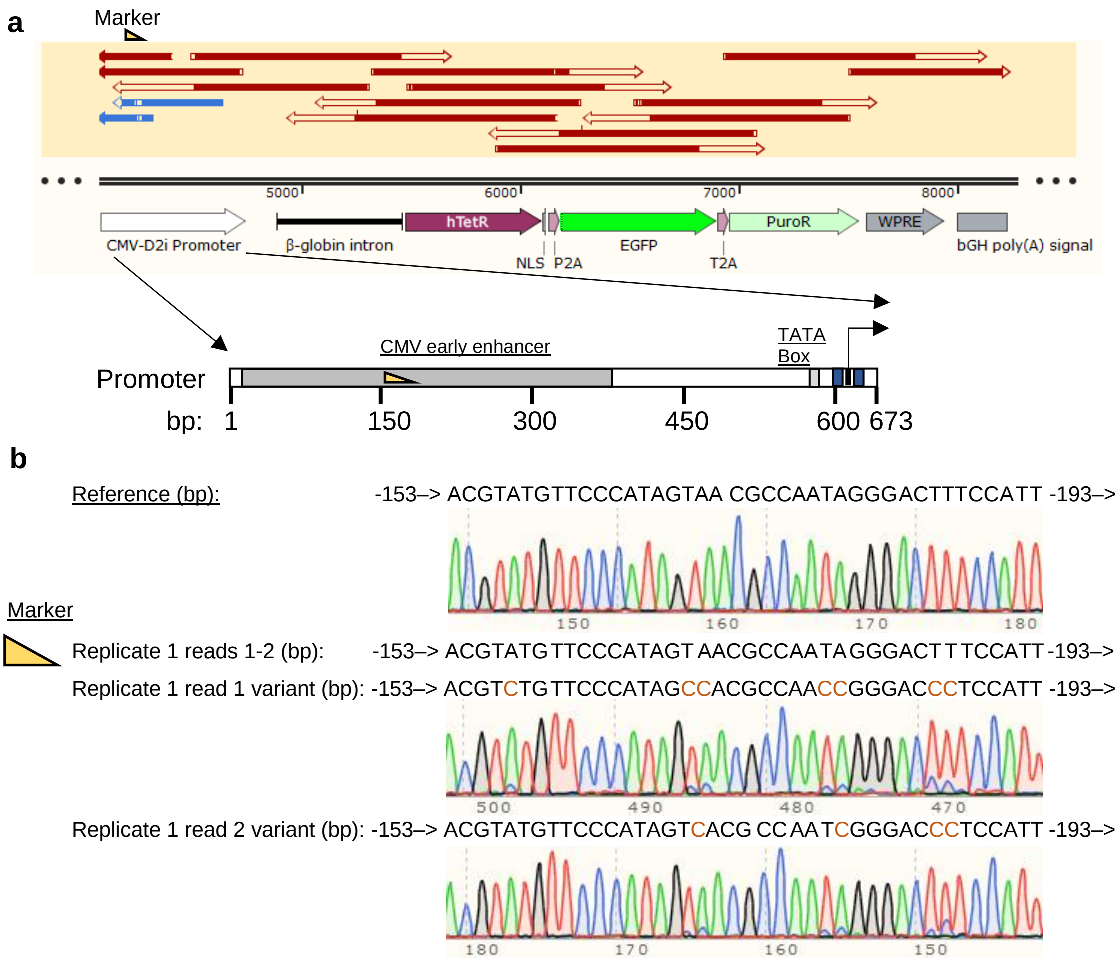

Supplementary Figure 25: Mutations in the CMV enhancer suggest genetic heterogeneity in the mNF-PuroR circuit from replicate 1 after 35 µg/mL Puromycin treatment.
(a) Sequencing map (red reads) of the entire mNF-PuroR circuit from replicate 1 with a zoom-in of the CMV-D2ir promoter, which has two reads showing genetic heterogeneity (predicted variants shown as blue reads) from mixed sequencing traces as detected by CRISP-ID[9]. The markers indicate the location of the mutations. **(b)** Nucleotide sequence for the reference promoter (ancestral) and genetic variants at the vicinity of the heterogenous mutations (orange) detected by CRISP-ID from mixed peaks in sequencing traces.

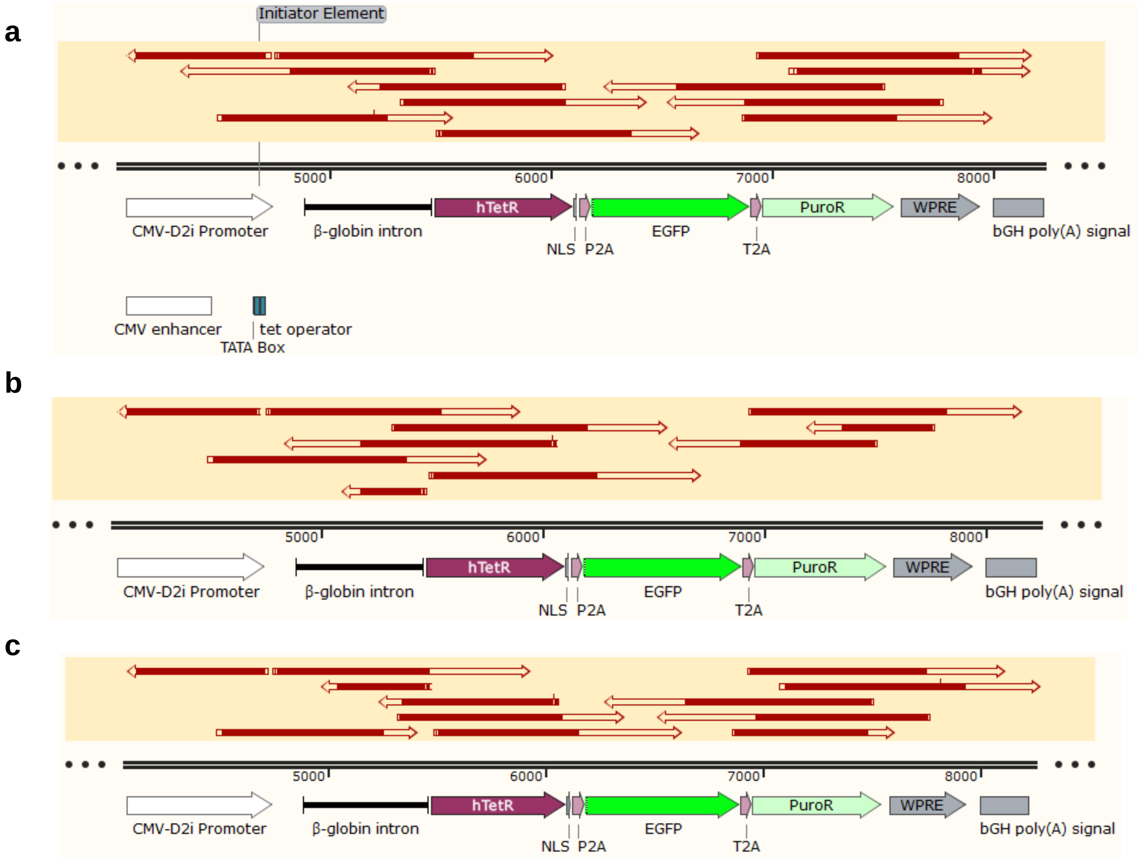

Supplementary Figure 26: Sequencing induced mNF-PuroR replicates 4 through 6 after removal of 35 µg/mL Puromycin treatment revealed no fixed mutations.
(a-c) Sequencing maps (red reads) of the induced mNF-PuroR circuit from **(a)** replicate 4, **(b)** replicate 5, and **(c)** replicate 6 previously under 35 µg/mL Puromycin treatment.

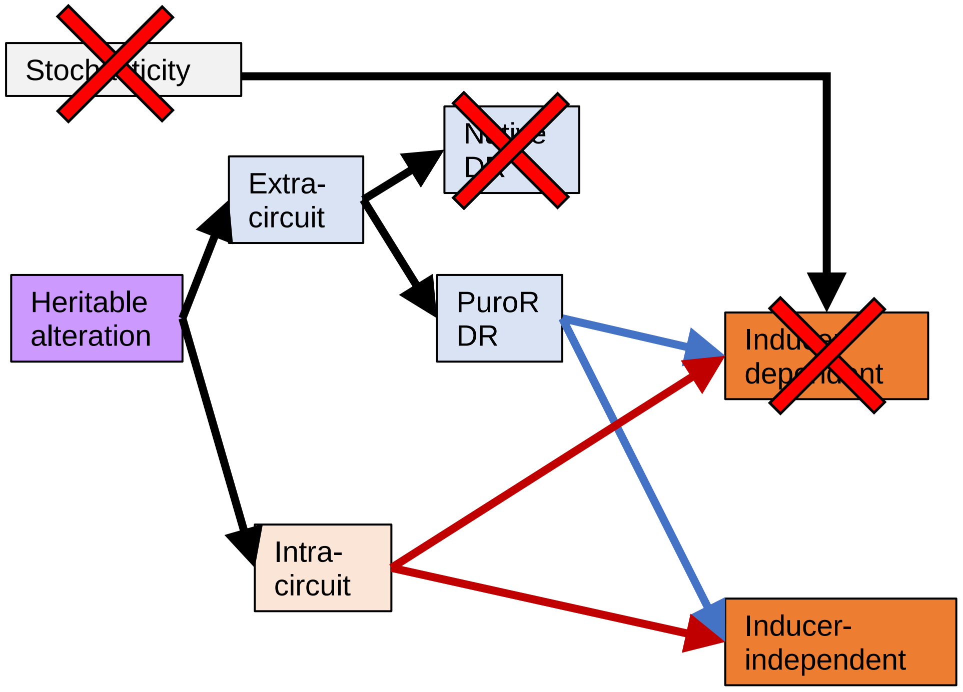

Supplementary Figure 27: mNF-PuroR at 35 μg/mL Puromycin adapts through inducer-independent genetic mechanisms.
We ruled out stochasticity at this Puromycin level because the uninduced and induced cells did not return to pre-induction and pre-treatment expression levels, respectively. This implies PuroR-dependent resistance and rules out extra-circuit native drug resistance. Sequencing results for mNF-PuroR replicates 1, 2, and 3 supports intra-circuit mutations leading to drug resistance. The remaining replicates do not contain fixed intra-circuit mutations, consistent with extra-circuit heritable drug resistance as an additional adaptation mechanism.

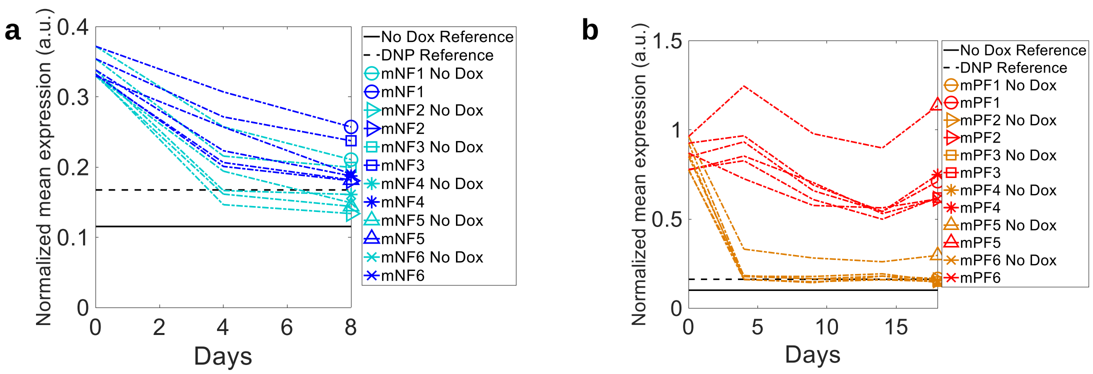

Supplementary Figure 28: Mean expression after temporary removal of 22.5 µg/mL Puromycin.

(a-b) Normalized mean expression of **(a)** mNF-PuroR and **(b)** mPF-PuroR after temporary removal of 22.5 µg/mL Puromycin. Uninduced mNF-PuroR or mPF-PuroR samples that were not treated nor induced (black solid line) and pre-treatment expression at the decoupled noise point (black dash line) served as references for expression reversion. Source data are provided as a Source Data file.

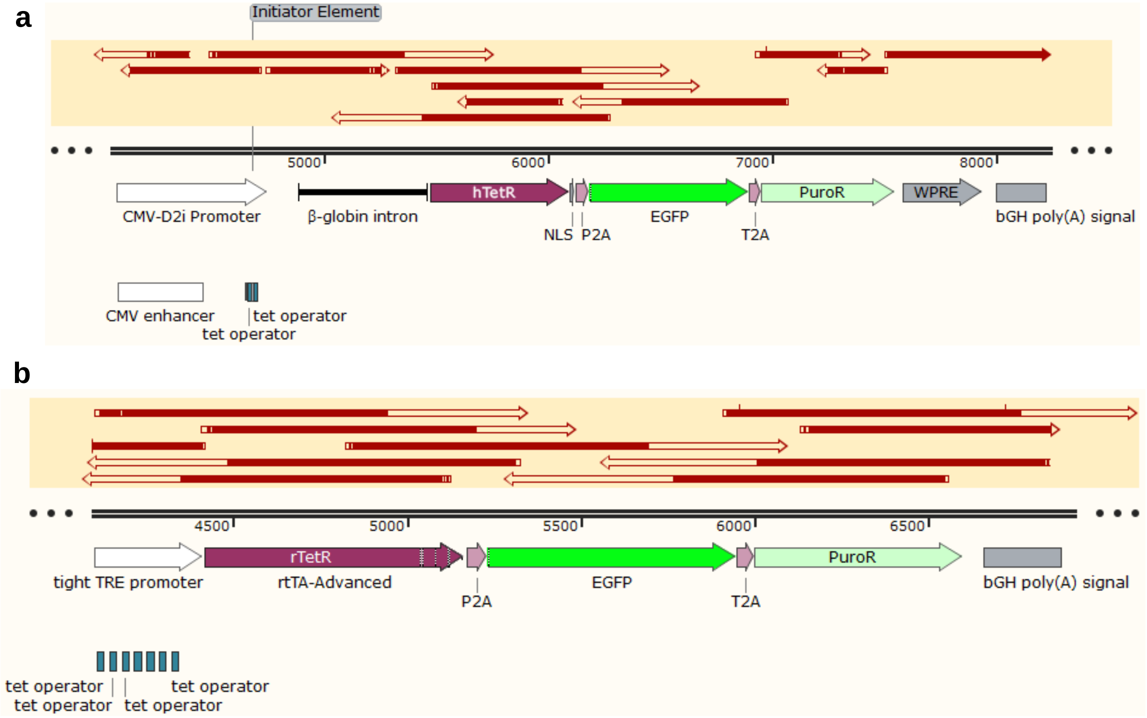

Supplementary Figure 29: The mNF-PuroR and mPF-PuroR circuit in cells from replicate 4 after removal of 22.5 µg/mL Puromycin did not harbor intra-circuit mutations.
(a) Sequencing results (red reads) for the mNF-PuroR circuit from cells in replicate 4 after removal of 22.5 µg/mL Puromycin. **(b)** Sequencing reads for the mPF-PuroR circuit from cells in replicate 4.

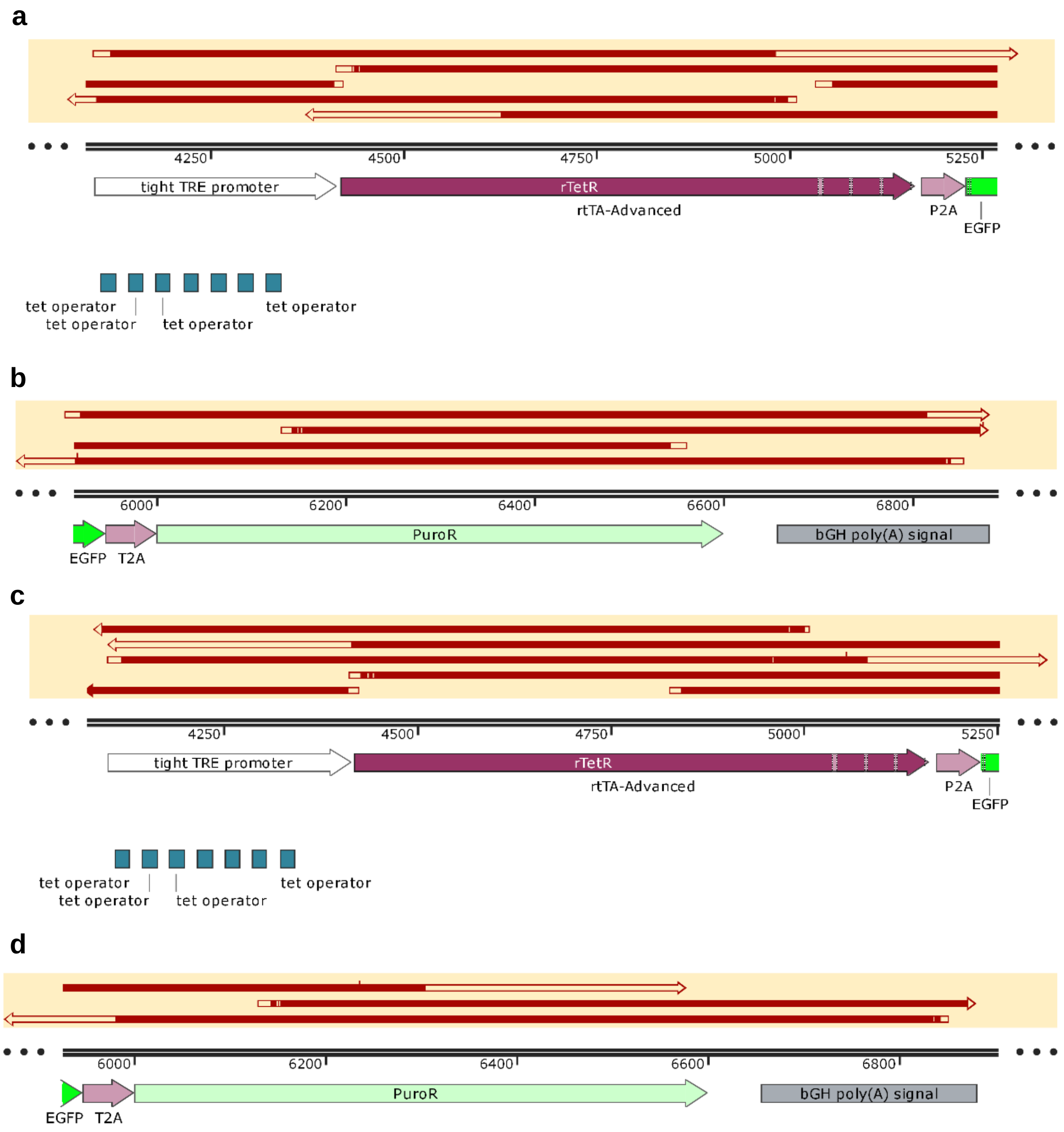

Supplementary Figure 30: Sequencing of induced mPF-PuroR circuits after removal of 35 µg/mL Puromycin indicated no mutations arose in the circuit.
(a) Sequencing reads (red arrows) for the pTRE promoter and *rtTA* regulator from induced mPF-PuroR replicate 2 after removing 35 µg/mL Puromycin. **(b)** Sequence reads of *PuroR* from mPF-PuroR replicate 2 circuit after removing 35 µg/mL Puromycin. **(c)** Sequencing reads for the pTRE promoter and *rtTA* regulator from mPF-PuroR replicate 3. **(d)** Sequencing results for the *PuroR* gene from mPF-PuroR replicate 3.

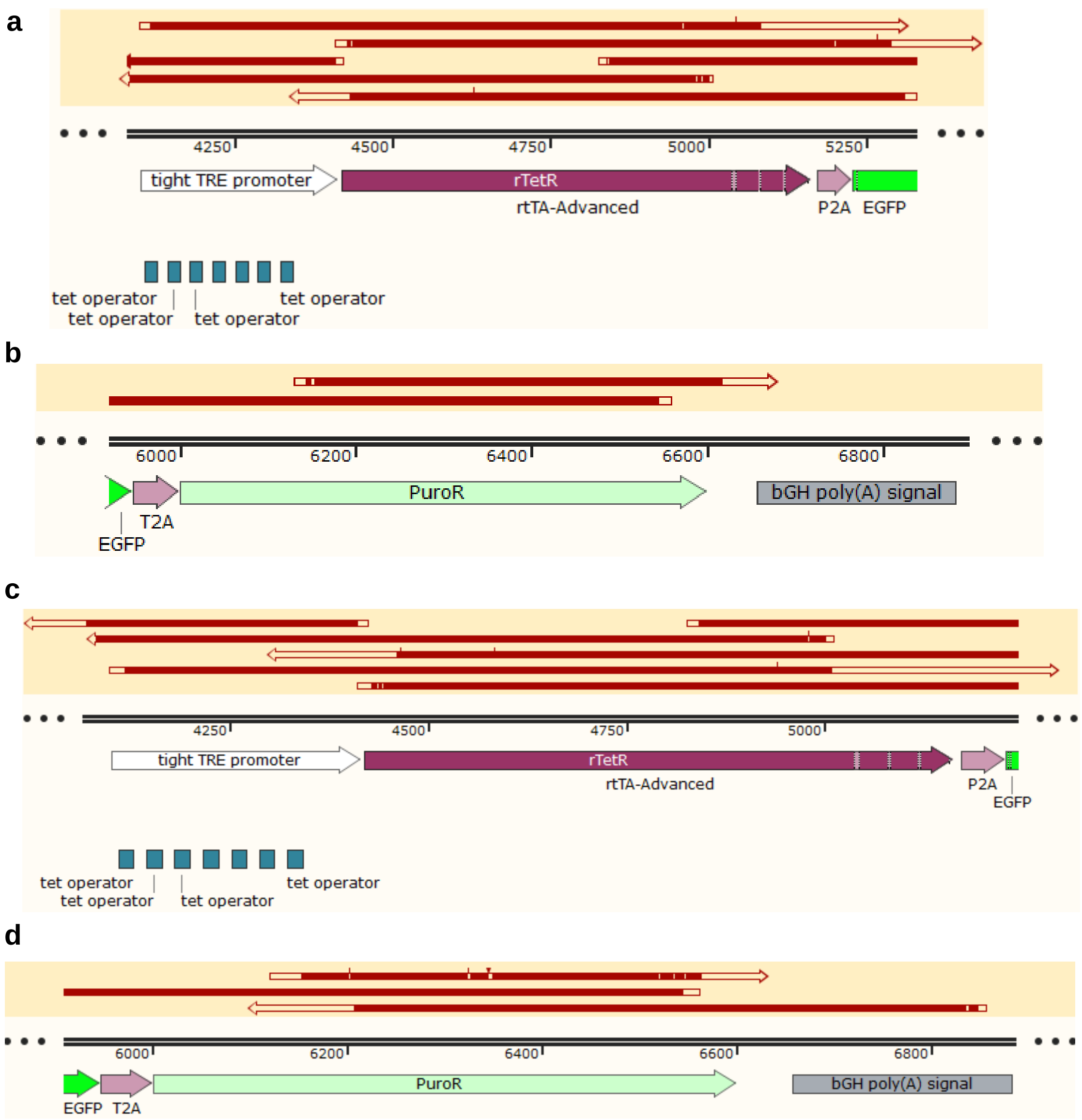

Supplementary Figure 31: Sequencing of induced mPF-PuroR replicates 3 and 4 circuits after removal of 50 µg/mL Puromycin did not reveal mutations in the circuits. **(a)** Sequencing results (red reads) for the pTRE promoter and *rtTA* regulator from the induced mPF-PuroR replicate 3 circuit after removal of 50 µg/mL Puromycin. **(b)** Sequencing reads for *PuroR* gene from mPF-PuroR replicate 3. **(c)** Sequencing results for the pTRE promoter and *rtTA* regulator from the induced mPF-PuroR circuit replicate 4 after removal of 50 µg/mL Puromycin. **(d)** Sequencing results for mPF-PuroR replicate 4 *PuroR* gene.

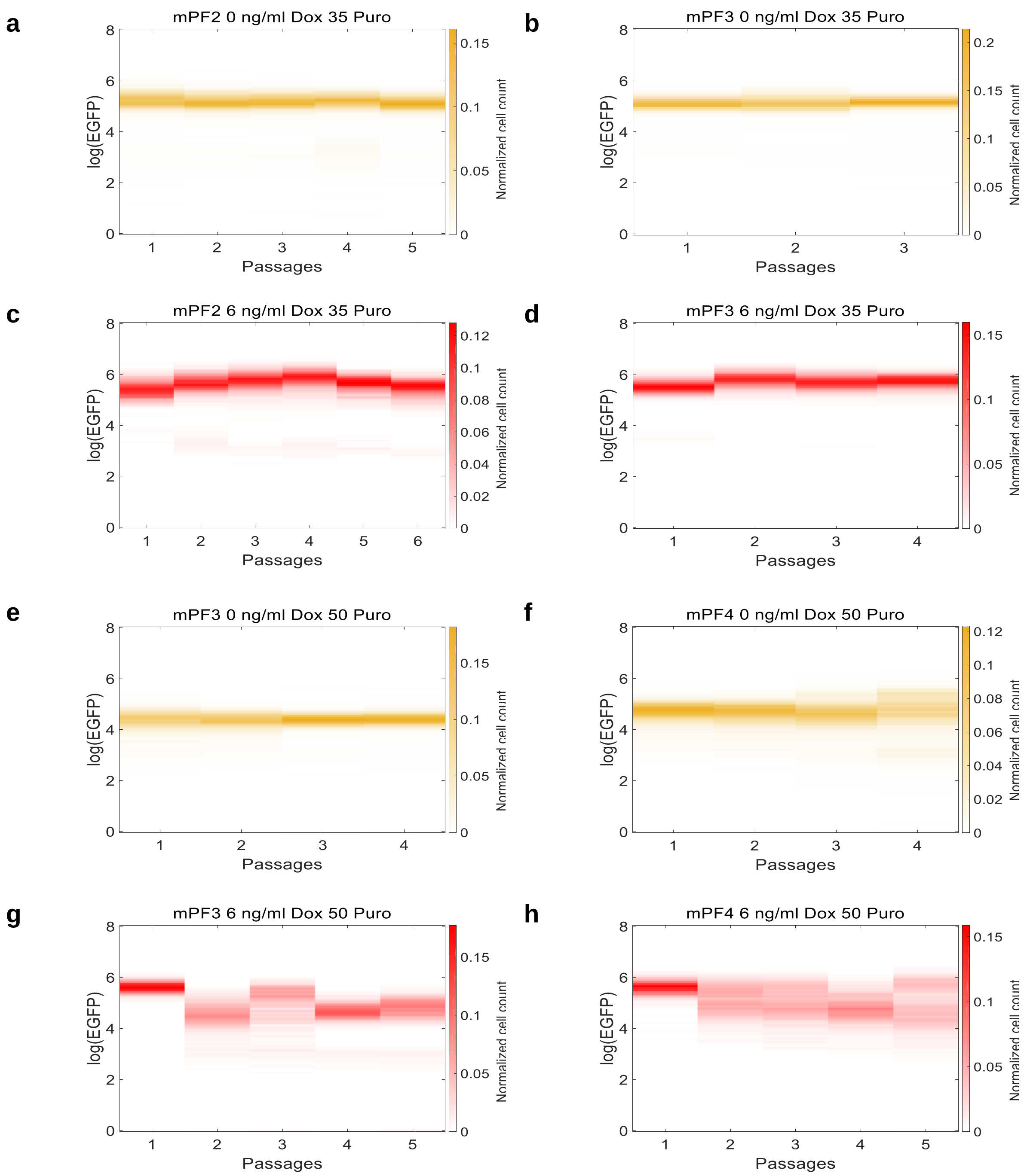

Supplementary Figure 32: Expression distributions of mPF-PuroR after removal of 35 and 50 µg/ml Puromycin.

(a-b) Log-transformed expression distributions for uninduced mPF-PuroR replicates 2-3 after removing 35 µg/mL Puromycin. **(c-d)** Log-transformed expression distributions for induced mPF-PuroR replicates 2-3 after removing 35 µg/mL Puromycin. **(e-f)** Expression distributions for uninduced mPF-PuroR replicates 3-4 after removing 50 µg/mL Puromycin. **(g-h)** Single-cell expression distributions for induced mPF-PuroR replicates 3-4 after removing 50 µg/mL Puromycin. Each color bar represents normalized cell counts going from low (white) to high (gold or red depending on induction).

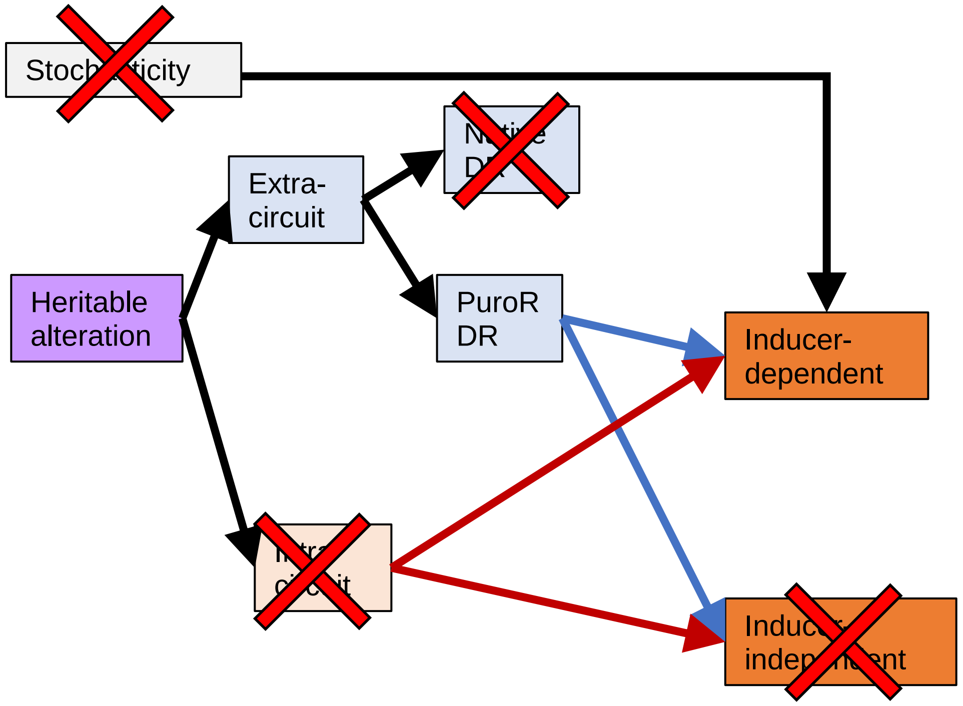

Supplementary Figure 33: Populations with mPF-PuroR at 35 and 50 µg/mL Puromycin adapt through extra-circuit inducer-dependent heritable mechanisms.
We ruled out stochasticity out because the cells did not return to pre-treatment expression levels. This also implies *PuroR*-dependent resistance and rules out extra-circuit native drug resistance. Sequencing results for mPF-PuroR replicates 2 and 3 from 35 µg/mL Puromycin and replicates 3 and 4 from 50 µg/mL Puromycin did not reveal intra-circuit mutations. Expression dropped after removal of Doxycycline. Therefore, inducer-dependent extra-circuit heritable drug resistance is the mechanism for mPF adaptation at these stress levels.

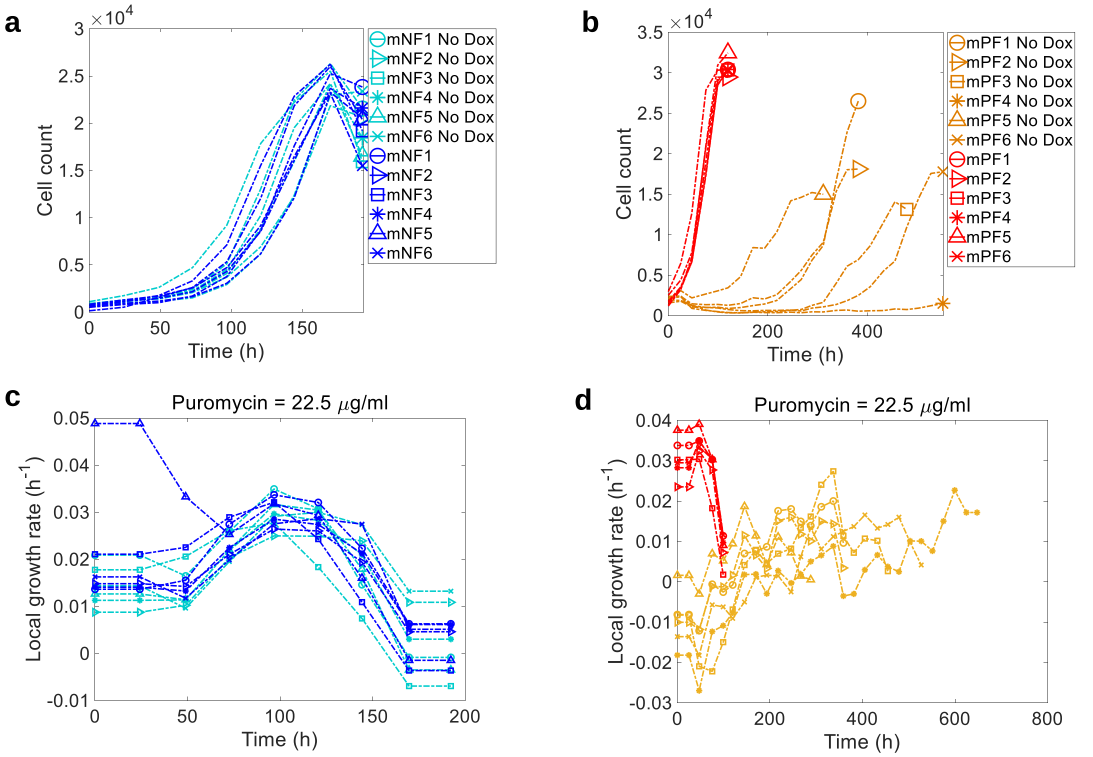

Supplementary Figure 34: Final treatment under 22.5 µg/mL Puromycin.
(a-b) Growth curves for **(a)** mNF-PuroR and **(b)** mPF-PuroR replicates retreated with 22.5 µg/mL Puromycin. **(c-d)** Local growth rates for **(c)** mNF-PuroR and **(d)** mPF-PuroR retreated with 22.5 µg/mL Puromycin. Each line plot is a replicate. Source data are provided as a Source Data file.

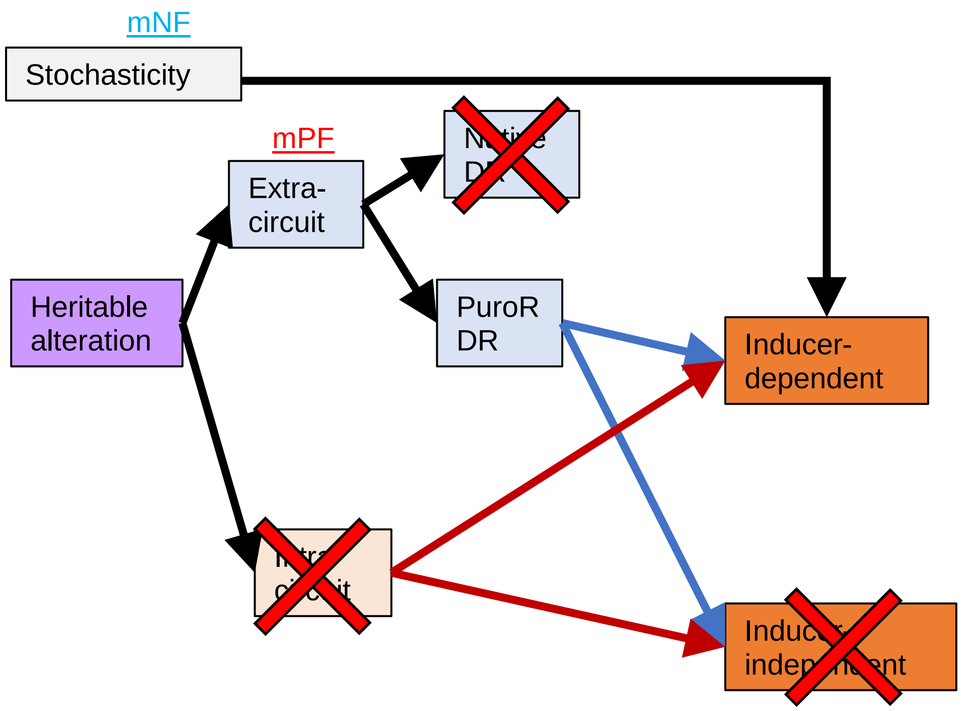

Supplementary Figure 35: Populations with mNF-PuroR at 22.5 μg/mL Puromycin adapt through stochastic mechanisms while mPF-PuroR cells adapt through extra-circuit heritable alterations in an inducer-dependent manner.

Stochasticity cannot be ruled out for cells with the low-noise mNF-PuroR circuit because, after drug removal, expression of both induced and uninduced cells dropped to pre-treatment and pre-induction levels, respectively. On the other hand, the induced mPF-PuroR populations maintained their expression above pre-treatment levels, which is consistent with extra-circuit heritable mechanisms. Accordingly, sequencing results for mNF-PuroR and mPF-PuroR replicate 4 revealed no intra-circuit mutations.

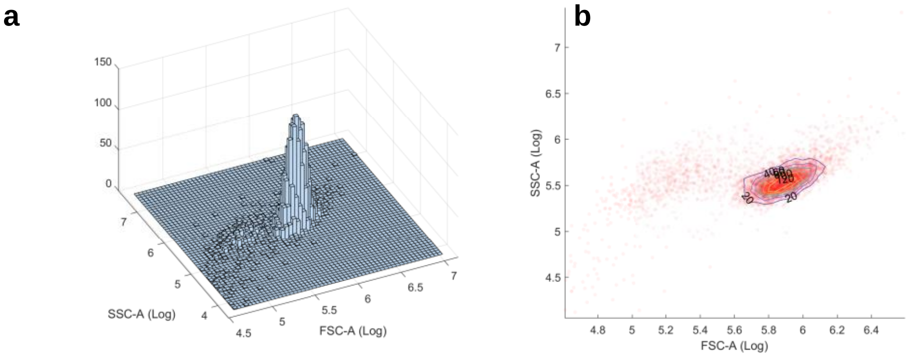

Supplementary Figure 36: Example of the density gating method for flow cytometry data.
(**a**) Example of the two-dimensional histograms from the density gating method. (**b**) Contour plot of the side scatter (SSC) and forward scatter (FSC) gate as a function of density binning.

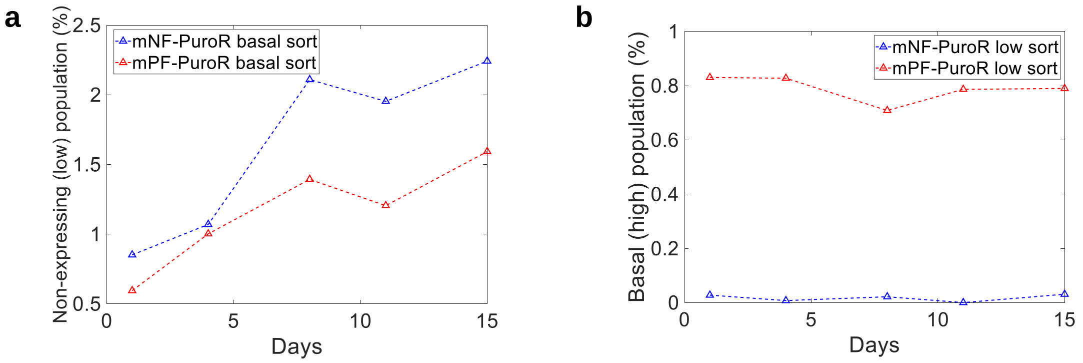

Supplementary Figure 37: FACS-sorting of non-expressing and basal subpopulations revealed no switching from the non-expressing state.
(a) Percent fraction of the non-expressing subpopulation over time after sorting for cells with basal (uninduced) expression levels. **(b)** Percent fraction of the basal-expressing subpopulation over time after sorting for the non-expressing subpopulation. Source data are provided as a Source Data file.

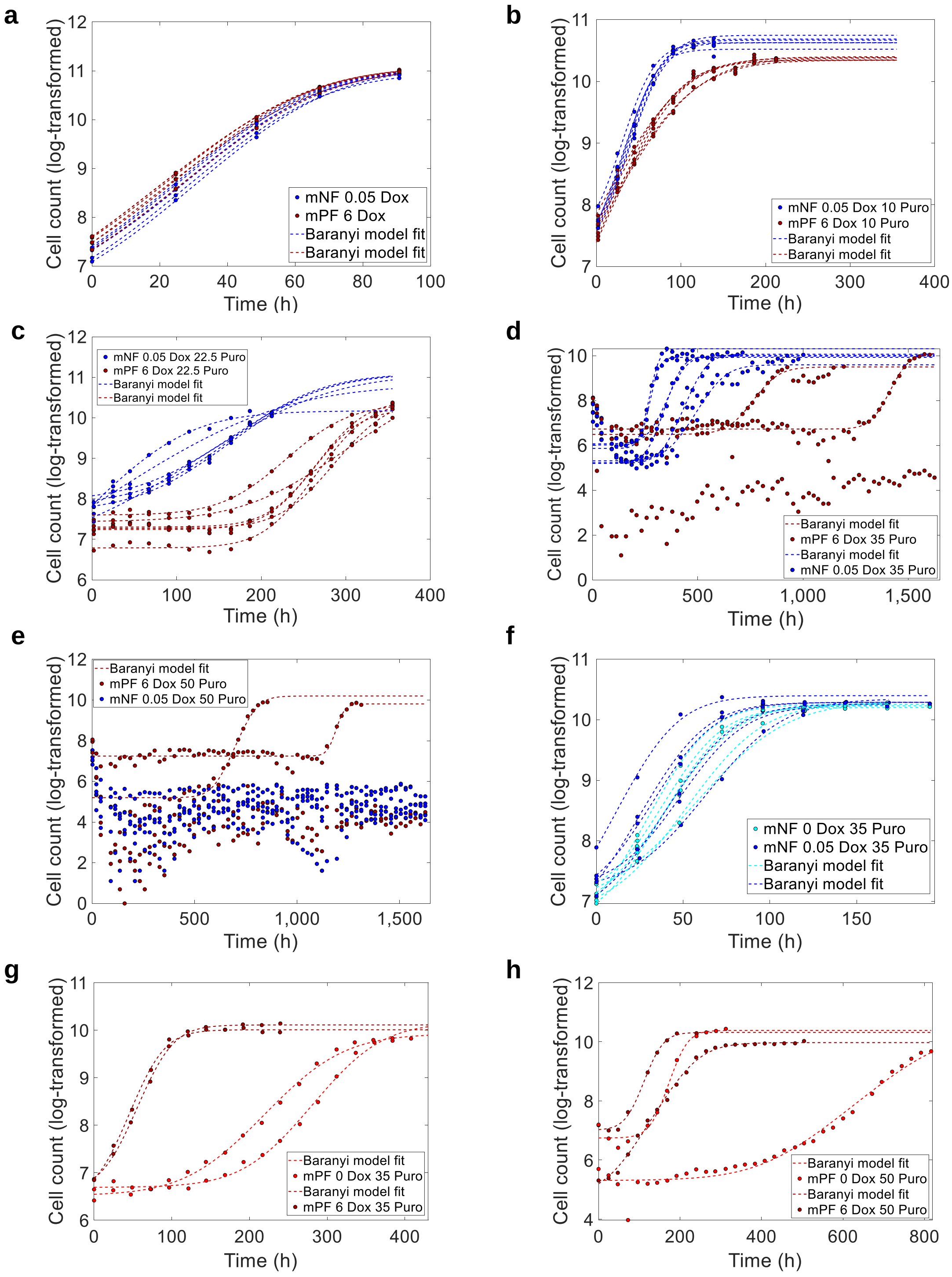

Supplementary Figure 38: Baranyi fits for the growth curves.
(a-e) Baranyi model growth curve fits for 0, 10, 22.5, 35, and 50 µg/mL Puromycin treatment phase. **(f-h)** Re-treatment phase fits for mNF-PuroR and mPF-PuroR under 35 µg/mL Puromycin, and mPF-PuroR under 50 µg/mL Puromycin. Each line plot is a replicate. Source data are provided as a Source Data file.

SUPPLEMENTARY TABLES

Sorted sample	Parameter	Value (day^{-1})
High-sorted mNF	f = r	2.776
Low-sorted mNF	f = r	2.663
High-sorted mPF	f = r	0.4142
Low-sorted mPF	f = r	0.3343

Supplementary Table 1: Flow-sort subpopulation fitting of switching rates.
Fall (f) and rise (r) switching rates were fitted with the H(t) solution for the high-expressing fraction in Supplementary Equation 1.

Function/Simulation Parameters	Strain	Symbol	Value
f(EGFP) and $A_{DmPF,mNF}$, Equations (3) & (4) in Methods			
	mPF	μ	4.4094
		σ	0.3835
	mNF	μ	4.5452
		σ	0.1774
A_{Dthres}, Equation (5) in Methods			
		β	5.1
		K	4
$A_{PmPF,mNF}$, Equation (6) in Methods			
	mPF	μ'	3.4556
		σ'	0.2599
	mNF	μ'	3.3176
		σ'	0.2392
Population Model			
		μ	0.0556
		η_1	$[1, 5, 4, 10, 10]\times10^{2}$
		η_2	$[1, 5, 2.5, 10, 5]\times10^{2}$
	mPF	N_0	$[0.1, 0.15, 0.2, 0.1, 0.1]\times10^{4}$
		N_{max}	$[6, 3, 3.25, 2.5, 2]\times10^{4}$
		$r_{P,N}$	$[1, 1, 1, 1, 1]\times10^{-4}$
		$r_{N,P}$	$[1, 1, 1, 1, 1]\times10^{-2}$
		$r_{G,N}$	$[0, 1, 1, 1, 1]\times10^{-7}$
		k_N	$[1, 0.825, 0.6, 0.45, 0.4]\times\mu$
		g_N	$[1, 2.5, 3, 3.5, 5]\times10^{-2}$
		$r_{G,P}$	$[0, 5, 5, 5, 5]\times10^{-6}$
		k_G	$[1, 0.825, 0.8, 0.45, 0.4]\times\mu$
		g_G	$[1, 1, 1, 1, 1]\times10^{-2}$
	mNF	N_0	$[0.085, 0.2, 0.25, 0.1, 0.1]\times10^{4}$
		N_{max}	$[6, 4, 3, 2.25, 2]\times10^{4}$
		$r_{P,N}$	$[1, 1, 1, 1, 1]\times10^{-4}$
		$r_{N,P}$	$[1, 1, 1, 1, 1]\times10^{-2}$
		$r_{G,N}$	$[0, 1, 1, 1, 1]\times10^{-6}$
		k_N	$[1, 0.825, 0.8, 0.45, 0]\times\mu$
		g_N	$[1, 2.5, 3, 3.5, 5]\times10^{-2}$
		$r_{G,P}$	$[0, 5, 5, 5, 5]\times10^{-6}$
		k_G	$[1, 0.825, 0.8, 0.45, 0.4]\times\mu$
		g_G	$[1, 1, 1, 1, 1]\times10^{-2}$

Supplementary Table 2: Parameters for stochastic population dynamics model.
Parameters provided in an array correspond to increasing Puromycin concentrations ([0 10 22.5 35 50] µg/ml). Single parameter values indicate the value used for all Puromycin treatment conditions. The same parameters were used for mPF and mNF strains unless otherwise indicated.

Primer name	Sequence (5' -> 3')
CMV-SpeI-f	GCGCACTAGTTATTAATAGTAATCAATTACG
rtTA-XhoI-adv-r	GCGCCTCGAGTTACCCGGGGAGCATGTCAAGGTC
CMV-PacI-f	GCGCTTAATTAATGACATTGATTATTGACTAGTTATTAATAG
SV40-AscI-BbvCI-r	CAGAAGGCTGAGGTAGCGGCGCGCCCCATAGAGCCCACCGCATCCCCAGC
TN-P2A-r	GCTGAAGTTAGTAGCTCCGCTTCCCTTTCTCTTCTTTTTTGGCCCGCCGC
P2A-TN-f	GCGGCGGGCCAAAAAAGAAGAGAAAGGGAAGCGGAGCTACTAACTTCAGC
P2A-i2-f	GGAAGCGGAGCTACTAACTTCAGCCTGCTGAAGCAGGCTGGAGACGTGGA
P2A-i1-EGFP-f	CTGCTGAAGCAGGCTGGAGACGTGGAGGAGAACCCTGGACCTATGGTGAG
EGFP-P2A-f	GGAGGAGAACCCTGGACCTATGGTGAGCAAGGGCGAGGAGCTG
pTRE-Tight-SpeI-f	GCGCACTAGTCGAGGCCCTTTCGTCTTCA
2nd-OL-pTRE-hrtTA-r	CTTTGCTCTTGTCCAGTCTAGACATTCCAGGCGATCTGACGGTTCACTAA
2nd-OL-pTRE-hrtTA-f	TTAGTGAACCGTCAGATCGCCTGGAATGTCTAGACTGGACAAGAGCAAAG
hrtTA-SalI-r	AAAAGTCGACTTACCCGGGGAGCATGTCAAG
pTRE-Tight-MluI-f	GCGCACGCGTCGAGGCCCTTTCGTCTTCA
pTRE-rtTA-OL-2A-r	CAGCAGGCTGAAGTTAGTAGCTCCGCTTCCCCCGGGGAGCATGTCAA
2A-OL-hEGFP-f	GCTGGAGACGTGGAGGAGAACCCTGGACCTATGGTGAGCAAGGGCGAGGA
2A-Bridge-OL-f	AGCTACTAACTTCAGCCTGCTGAAGCAGGCTGGAGACGTGGAGGAGAACC
hEGFP-BamHI-r	GCGCGGATCCTTACTTGTACAGCTCGTCCATGC
T2A-f	GAGGGCAGAGGAAGTCTTCTAACATG
NotI-AgeI-PuroR-r	GCGGCCGCACCGGTTCAGGCACCGGGCTTGCG
Intron-SbfI-f	CCTACAGGTCCTGCAGGCGCCAC
T2A-hEGFP-OL-r	AGGGCCGGGATTCTCCTCCACGTCACCGCATGTTAGAAGACTTCCTCTGCCC TCCTTGTACAGCTCGTCCATGCCG
SacI-pCMV-f	GCAGAGCTCGTTTAGTGAACCGT

Supplementary Table 3: List of primers used for plasmid construction.
Primers were diluted with molecular-grade water to a working stock of 10 µM from 100 µM frozen stocks.

Primer name	Sequence (5’ -> 3’)
D2ir-Enh-Seq-f	ACGGGCCAGATATACGCGTT
NF-Edge-f	GCGTTGACATTGATTATTGACTAGTT
FRT-Circuit-3-r	GCTGGTTCTTTCCGCCTCAG
MluI-pTRE-f	ATATACGCGTCGAGGCCCTTTC
hEGFP-130-qPCR-f	ACGACGGCAACTACAAGAC
bGH-pA-r	GCTGGTTGCTAAGAGGGAGG
FRT-Circuit-2-r	CGAACGTGGCGAGAAAG

Supplementary Table 4: List of primers for circuit sequencing.
Blue shaded rows contain primers ampifying the mNF-PuroR circuit, red rows indicate primers used for amplifying the mPF-PuroR circuit, and primers in purple rows were used for both circuits.

Primer name	Sequence (5’ -> 3’)
PuroR-150-qPCR-f	CGAGTACAAGCCCACGGT
PuroR-150-qPCR-r	AGTTCTTGCAGCTCGGTGA
Vinculin fwd	GCTGGTTGCTAAGAGGGAGG
Vinculin rev	ATCAGAGGCAGCTTTCACGG

Supplementary Table 5: List of primers used for qPCR copy number analysis.
Primers were diluted with molecular-grade water to a working stock concentration of 10 µM from 100 µM frozen stocks. The *Vinculin* primer sequences were obtained from the literature[10].

SUPPLEMENTARY REFERENCES

1 Nevozhay, D., Zal, T. & Balázsi, G. Transferring a synthetic gene circuit from yeast to mammalian cells. *Nature Communications* **4**, 1451, doi:10.1038/ncomms2471 (2013).

2 Nevozhay, D., Adams, R. M., Murphy, K. F., Josic, K. & Balázsi, G. Negative autoregulation linearizes the dose-response and suppresses the heterogeneity of gene expression. *Proceedings of the National Academy of Sciences of the United States of America* **106**, 5123–, doi:10.1073/pnas.0809901106 (2009).

3 Nevozhay, D., Adams, R. M., Van Itallie, E., Bennett, M. R. & Balázsi, G. Mapping the environmental fitness landscape of a synthetic gene circuit. *PLOS Computational Biology* **8**, e1002480, doi:10.1371/journal.pcbi.1002480 (2012).

4 Belete, M. K. & Balazsi, G. Optimality and adaptation of phenotypically switching cells in fluctuating environments. *Phys Rev E* **92**, doi:10.1103/PhysRevE.92.062716 (2015).

5 Xu, X. *et al.* The genomic sequence of the Chinese hamster ovary (CHO)-K1 cell line. *Nature Biotechnology* **29**, 735-U131, doi:10.1038/nbt.1932 (2011).

6 Kaern, M., Elston, T. C., Blake, W. J. & Collins, J. J. Stochasticity in gene expression: from theories to phenotypes. *Nature reviews. Genetics* **6**, 451–464, doi:10.1038/nrg1615 (2005).

7 Blake, W. J. *et al.* Phenotypic consequences of promoter-mediated transcriptional noise. *Molecular cell* **24**, 853–865, doi:10.1016/j.molcel.2006.11.003 (2006).

8 Isackson, P. J. & Bertrand, K. P. Dominant negative mutations in the Tn10 Tet repressor - evidence for use of the conserved helix-turn-helix motif in DNA-binding. *Proceedings of the National Academy of Sciences of the United States of America* **82**, 6226-6230, doi:DOI 10.1073/pnas.82.18.6226 (1985).

9 Dehairs, J., Talebi, A., Cherifi, Y. & Swinnen, J. V. CRISP-ID: decoding CRISPR mediated indels by Sanger sequencing. *Scientific Reports* **6**, 28973, doi:10.1038/srep28973 (2016).

10 Lee, J. S., Kallehauge, T. B., Pedersen, L. E. & Kildegaard, H. F. Site-specific integration in CHO cells mediated by CRISPR/Cas9 and homology-directed DNA repair pathway. *Scientific Reports* **5**, 8572, doi:10.1038/srep08572 (2015).

11 Gillespie, D. T. Exact numerical simulation of the Ornstein-Uhlenbeck process and its integral. *Phys Rev E* **54**, 2084-2091, doi:DOI 10.1103/PhysRevE.54.2084 (1996).

12 Charlebois, D. A., Abdennur, N. & Kaern, M. Gene expression noise facilitates adaptation and drug resistance independently of mutation. Physical Review Letters **107**, doi:10.1103/PhysRevLett.107.218101 (2011).

13 Charlebois, D. A., Intosalmi, J., Fraser, D. & Kaern, M. An algorithm for the stochastic simulation of gene expression and heterogeneous population dynamics. Communications in Computational Physics **9**, 89-112, doi:10.4208/cicp.280110.070510a (2011).
