# Supplementary material for: Role of network-mediated stochasticity in mammalian drug resistance
Source: Nat Commun. 2019 Jun 24;10:2766. doi: 10.1038/s41467-019-10330-w (PMC6591227; doi:10.1038/s41467-019-10330-w)
Supplement: Supplementary file 3 — Reporting Summary [file 41467_2019_10330_MOESM3_ESM.pdf]

nature research

Corresponding author(s): Gábor Balázsi

Last updated by author(s): Apr 18, 2019

Reporting Summary

Nature Research wishes to improve the reproducibility of the work that we publish. This form provides structure for consistency and transparency in reporting. For further information on Nature Research policies, see Authors & Referees and the Editorial Policy Checklist.

Statistics

For all statistical analyses, confirm that the following items are present in the figure legend, table legend, main text, or Methods section.

n/a	Confirmed	
☐	☒	The exact sample size (*n*) for each experimental group/condition, given as a discrete number and unit of measurement
☐	☒	A statement on whether measurements were taken from distinct samples or whether the same sample was measured repeatedly
☐	☒	The statistical test(s) used AND whether they are one- or two-sided<br>*Only common tests should be described solely by name; describe more complex techniques in the Methods section.*
☐	☒	A description of all covariates tested
☐	☒	A description of any assumptions or corrections, such as tests of normality and adjustment for multiple comparisons
☐	☒	A full description of the statistical parameters including central tendency (e.g. means) or other basic estimates (e.g. regression coefficient) AND variation (e.g. standard deviation) or associated estimates of uncertainty (e.g. confidence intervals)
☐	☒	For null hypothesis testing, the test statistic (e.g. *F*, *t*, *r*) with confidence intervals, effect sizes, degrees of freedom and *P* value noted<br>*Give P values as exact values whenever suitable.*
☒	☐	For Bayesian analysis, information on the choice of priors and Markov chain Monte Carlo settings
☒	☐	For hierarchical and complex designs, identification of the appropriate level for tests and full reporting of outcomes
☒	☐	Estimates of effect sizes (e.g. Cohen's *d*, Pearson's *r*), indicating how they were calculated

Our web collection on statistics for biologists contains articles on many of the points above.

Software and code

Policy information about availability of computer code

Data collection	We used Nikon Elements AR version 4.51.01 to acquire images. The BD Accuri C6 Software was used to acquire flow data.
Data analysis	We used MATLAB r2018b to analyze the flow cytometry data and growth curve data. We used Nikon Elements AR version 4.51.01 to analyze the imaging data. The plasmid editor SnapGene 3.1.4 was used to design plasmid construction and align sequencing traces. We used the CRISP-ID algorithm version 1.1a to extract subpopulation sequences from mixed sequencing traces.

For manuscripts utilizing custom algorithms or software that are central to the research but not yet described in published literature, software must be made available to editors/reviewers. We strongly encourage code deposition in a community repository (e.g. GitHub). See the Nature Research guidelines for submitting code & software for further information.

Data

Policy information about availability of data

All manuscripts must include a data availability statement. This statement should provide the following information, where applicable:

- Accession codes, unique identifiers, or web links for publicly available datasets
- A list of figures that have associated raw data
- A description of any restrictions on data availability

The raw growth curve data for Figs. 5c-g, 7e-g; DNA sequences for the plasmids shown in Figs. 2a, 3a, Supplementary Figures 4a, 6a; ab1 sequencing traces with associated template DNA sequence files for Supplementary Figures 23-26, 29-31; and flow cytometry data for Figs. 2b-d, 3b-d, 4a-c, 7b-d, and associated Supplementary figures can be found at https://openwetware.org/wiki/CHIP:Data. The remaining data supporting the findings in the study are available from the corresponding author, G.B., upon reasonable request. Source data are included as a separate source file.

Field-specific reporting

Please select the one below that is the best fit for your research. If you are not sure, read the appropriate sections before making your selection.

☒ Life sciences ☐ Behavioural & social sciences ☐ Ecological, evolutionary & environmental sciences

For a reference copy of the document with all sections, see nature.com/documents/nr-reporting-summary-flat.pdf

Life sciences study design

All studies must disclose on these points even when the disclosure is negative.

Sample size	For samples in the drug treatment experiment intended for statistical analysis with the non-parametric two-tailed Mann-Whitney U test, we chose a sample size of six to trade-off the power of the test with n = 6 with the practicality of handling six replicates during long-term (3-6 months) imaging, flow cytometry, and circuit sequencing. For the circuit dose-responses, we ran experiments in triplicate (n = 3), which did not require statistical testing.
Data exclusions	During the drug treatment experiment, mPF-PuroR replicates 4, 5 and 6 under 35 micrograms per ml Puromycin and mPF-PuroR replicates 5 and 6 under 50 micrograms per ml Puromycin became contaminated. The data from these replicates had to be excluded. During the flow sorting experiments to estimate cellular memory, there were samples at particular time points that were outliers; the fluorescence values had no biological value. These time points were excluded from the mNF-PuroR and mPF-PuroR memory experiments.
Replication	The drug treatment experiments and flow cytometry dose-responses in this study are replicate experiments from previous work we completed testing the same hypothesis (unpublished).
Randomization	For the flow cytometry and drug treatment experiments, samples were distributed into replicate groupings based on the position of teach sample in 24-well plates, with replicate 1 on the left-top most portion of the plate, and replicates 2...N going rightward in each row.
Blinding	Blinding was not relevant in this study because there were no human subjects in our study. Additionally, blinding would be impractical when sequencing replicates that have an unknown circuit (during blinding), which must be unblinded once the circuit is identified. Again, we did not need blinding in this experiment.

Reporting for specific materials, systems and methods

We require information from authors about some types of materials, experimental systems and methods used in many studies. Here, indicate whether each material, system or method listed is relevant to your study. If you are not sure if a list item applies to your research, read the appropriate section before selecting a response.

Materials & experimental systems

n/a	Involved in the study	
☒	☐	Antibodies
☐	☒	Eukaryotic cell lines
☒	☐	Palaeontology
☒	☐	Animals and other organisms
☒	☐	Human research participants
☒	☐	Clinical data

Methods

n/a	Involved in the study	
☒	☐	ChIP-seq
☐	☒	Flow cytometry
☒	☐	MRI-based neuroimaging

Eukaryotic cell lines

Policy information about cell lines

Cell line source(s)	Invitrogen provided the Flp-In CHO cell line (Catalog number: R75807)
Authentication	The Flp-In CHO cells obtained from Invitrogen were authenticated prior to purchase.
Mycoplasma contamination	We did not see any indicators of mycoplasma contamination.
Commonly misidentified lines (See ICLAC register)	The CHO cell line is not listed as commonly misidentified in the ICLAC register.

Flow Cytometry

Plots

Confirm that:

☐ The axis labels state the marker and fluorochrome used (e.g. CD4-FITC).

☒ The axis scales are clearly visible. Include numbers along axes only for bottom left plot of group (a 'group' is an analysis of identical markers).

☐ All plots are contour plots with outliers or pseudocolor plots.

☒ A numerical value for number of cells or percentage (with statistics) is provided.

Methodology

Sample preparation	Before measuring expression, cell samples were trypsinized, neutralized with media, centrifuged at 300 x g for 5 minutes, resuspended in 1X DPBS, and then strained into a 5 mL polystyene round-bottom tubed (VWR, 21008948) for subsequent flow cytometry data collection.
Instrument	BD Accuri C6 benchtop flow cytometer measured EGFP fluorescence data from single CHO cells. Cells were sorted by the FACSAria III instrument. For estimating relaxation of expression for cells sorted for high or low fluorescence, we collected flow data with the BD FACSCalibur Cell Analyzer.
Software	We used the BD Accuri C6 Software for flow data collection. Flow data was analyzed with custom MATLAB scripts. See https://openwetware.org/wiki/CHIP:Data to obtain the scripts.
Cell population abundance	Up to 20,000 events were gated for analysis. Samples treated with drug typically had lower cell counts (>500).
Gating strategy	We developed custom MATLAB scripts to gate and analyze flow cytometry data. Cells were adaptively gated with a density-threshold fit of log-transformed SSC and FSC values per sample to exclude debris. Specifically, flow cytometry data was exported as individual FCS files, which were then analyzed by custom MATLAB scripts. Raw forward and side scatter values were log-transformed, and then plotted as a 2-dimensional histogram. The region of greatest density was gated. The 2-dimensional histogram counts were then plotted as a contour, which further subdivided the plot with a density gate. We chose the second from the widest contour level on average, which increased in density at the SSC-FSC coordinates harboring cellular events.

☒ Tick this box to confirm that a figure exemplifying the gating strategy is provided in the Supplementary Information.
